# From Product-Quality Complaints to Patient-Safety Triage: A Structured Narrative Review and Proposed PRCS-TRACE Framework

**DOI:** 10.3390/healthcare14162597

**Published:** 2026-08-18

**Authors:** Sabihe Cenaj, Erand Llanaj

**Affiliations:** 1 Faculty of Medicine, University of Medicine, 1005 Tirana, Albania; 2 Working Group Translational Research and Health Services Research in General Practice, Medical Faculty OWL, Bielefeld University, 33615 Bielefeld, Germany; 3 Department of General Practice and Family Medicine, Medical Faculty OWL, Bielefeld University, 33615 Bielefeld, Germany

**Keywords:** product-quality complaints, pharmacovigilance, patient safety, medication errors, signal detection, medication management, drug recalls, substandard and falsified medicines, pharmacoepidemiology, healthcare delivery

## Abstract

Product-quality complaints are usually handled as pharmaceutical quality-system events, yet defects in sterility, potency, identity, packaging, labelling, storage, distribution or delivery-device function may affect medication use, treatment continuity and patient outcomes. This structured narrative review examines product-quality complaints (PQCs) as a patient-safety interface linking pharmaceutical quality systems, pharmacovigilance, medication-error prevention, recall action and pharmacoepidemiology. Existing pharmacovigilance, quality-management and recall systems address different components of this pathway, but no integrated framework was identified in the sources reviewed that specifies how product-quality complaints should be linked to exposure evidence and patient outcomes. Sources were identified through targeted PubMed searches and purposive retrieval of official regulatory, pharmacovigilance and public-health documents up to 5 June 2026; the review was not registered and included no quantitative synthesis. The sources identified concentrate on regulatory architecture, sentinel contamination events and shortage-associated harms; routine complaints are studied comparatively little, and no source identified reported the complaint-to-defect-to-exposure-to-outcome cascade with a complaint-level denominator. We propose the term patient-relevant complaint status (PRCS) for a complaint warranting patient-safety triage because the reported defect could plausibly affect exposure, sterility, potency, identity, delivery, medication use or treatment continuity, together with a TRACE workflow, a six-level clinical-consequence classification and separately graded certainty for defect confirmation, patient exposure and outcome attribution. These tools are proposed, unvalidated and hypothesis-generating; they do not convert complaints into adverse reactions, recalls into causality or spontaneous reports into incidence. Their intended role is to structure patient-safety triage, batch-aware linkage, exposure reconstruction, clinical follow-up and proportionate mitigation while causal attribution remains incomplete; this role requires prospective validation before routine implementation.

## 1. Introduction

Product-quality complaints (PQCs) are often the first formal trace of a medicine-quality problem [1]. A patient observes visible particles in an injectable. A pharmacist identifies a wrong overwrap. A nurse reports a delivery component that fails during administration. A patient reports loss of effect after a refill. In quality systems, these events may be recorded as complaints [2,3,4,5], deviations or suspected defects. In clinical systems, the same chain may present as a near miss, medication error, treatment interruption, lack of efficacy, toxicity or adverse event [6,7,8,9,10].

The patient-safety question is not whether every complaint represents harm [11,12]. A complaint does not by itself establish a defect, exposure or clinical harm, and no complaint-level denominator was identified in this review, so the prevalence of patient-relevant defects among complaints is unknown. The relevant question is narrower and more consequential: whether the reported defect plausibly reached a patient and changed exposure, medication use, treatment continuity, monitoring or clinical outcome [13]. We describe complaints as upstream rather than early.

A complaint sits upstream of signal validation in the evidentiary chain. We make no claim that complaints predict harm, that they precede validated signals in time or that they have measurable predictive performance; no study identified in this review reports the complaint-to-defect-to-exposure-to-outcome cascade with a complaint-level denominator, and that absence is the principal evidence gap this review identifies. This question has global relevance because medicine-quality surveillance [14,15], pharmacovigilance capacity [1] and recall execution vary across health systems [16], while the clinical consequences of sterility failure, potency drift, labelling error, delivery malfunction, impurity exposure or shortage can cross jurisdictional boundaries.

Current systems often keep product-quality investigation, pharmacovigilance, medication-error reporting, recall management and pharmacoepidemiological evaluation in separate operational lanes. This separation is administratively understandable but clinically incomplete [17,18]. A defect may begin as a quality event, become a medication-use hazard, generate a spontaneous report, trigger a recall, interrupt treatment and eventually require outcome-based evaluation [19]. The scientific task is not to collapse these domains into one category, but to define the evidentiary transitions between them.

This review asks when a product-quality complaint should become patient relevant without being overinterpreted as proof of harm. This review aimed to synthesise evidence on pathways linking product-quality complaints to patient exposure and outcomes, and to propose a testable, batch-aware framework for deciding when such complaints warrant patient-safety assessment. The purpose of the proposed framework is therefore not to replace existing quality or pharmacovigilance processes, but to define a structured interface between them and to generate testable hypotheses for future validation.

We avoid the word signal in the name of the construct. In pharmacovigilance, a signal is information arising from one or more sources that suggests a new potentially causal association, or a new aspect of a known association, that warrants verifying action; that meaning is established in GVP Module IX and in WHO-UMC practice [20]. The state described here is upstream of that meaning: a complaint may be assigned this status before any defect is confirmed, before any adverse-event report exists and before any assessment of causality has begun. Using signal for both would collapse the distinction the article exists to make.

## 2. Materials and Methods

### 2.1. Design and Rationale

This article is a structured narrative review [21,22,23] that integrates regulatory, clinical, pharmacovigilance, medication-safety and pharmacoepidemiological material to clarify boundaries, identify evidence gaps and generate testable propositions. This approach was selected because the relevant evidence spans legal requirements, quality-system guidance, outbreak investigations, signal-management concepts, observational studies and operational patient-safety practice [24,25].

The review is not a systematic review, meta-analysis, regulatory guidance document, validated classification study or incidence analysis. Its aim is conceptual and translational: to define when the evidentiary states set out once, in Section 3, justify patient-safety action under uncertainty.

### 2.2. Scope

The review covered medicinal products, biologicals, packaging, labelling, container-closure systems, delivery components, storage and distribution conditions [1,13,16], field alerts, recalls, spontaneous safety reports, medication-error pathways and pharmacoepidemiological approaches to medicine-quality risk [25,26,27]. We excluded industrial quality-control topics without plausible patient exposure, theoretical manufacturing defects without clinical or regulatory relevance and adverse-event discussions lacking a product-quality component. Supply-disruption sequelae of quality events—recall-driven substitution, treatment interruption and shortage-associated harm—are included by design as a distinct, indirect pathway from defect to patient consequence, and are kept analytically separate from defects acting through altered product chemistry, sterility or delivery.

### 2.3. Source Identification

Sources were identified through targeted, non-systematic searches of PubMed and purposive retrieval of official regulatory, pharmacovigilance and public-health documents up to 5 June 2026 [21,22,23]. Sources included the US Food and Drug Administration (FDA), electronic Code of Federal Regulations, European regulatory and GMP framework, International Council for Harmonisation (ICH), World Health Organization (WHO), Medicines and Healthcare products Regulatory Agency (MHRA) Yellow Card Scheme, and European pharmacovigilance resources and Uppsala Monitoring Centre (UMC). Citation tracking was used to identify sentinel contamination events, recall and signal-management frameworks, and pharmacoepidemiological examples of defect-to-harm pathways.

Search concepts combined terms for product-quality complaints, quality defects, recalls, field alerts, pharmacovigilance, spontaneous reporting, signal detection, contamination, sterility, potency, medication errors, labelling, packaging, device malfunction, shortages, substandard and falsified medicines, pharmacoepidemiology and patient harm. The full narrative-review search transparency statement, source categories, PubMed search string and source-selection logic are provided in Supplementary S1.

Because the source-identification process was targeted and iterative rather than protocol-driven, no PRISMA 2020 study-selection flow diagram is provided [28]. Record counts, title-and-abstract screening counts and full-text assessment counts were not prospectively logged and were not reconstructed retrospectively. The review should therefore be interpreted as a structured, source-prioritised narrative synthesis rather than as an exhaustive incidence review, effectiveness review or quantitative evidence synthesis.

### 2.4. Evidence Appraisal and Synthesis

Evidence was weighted by inferential function rather than by a single hierarchy [22,23,24]. Primary regulatory texts and official guidance were used to define duties, categories and signal-management processes [2,20,29,30,31,32,33]. Peer-reviewed clinical, epidemiological and regulatory-science studies were used for empirical claims where exposure and outcome data were available [19,34,35]. Sentinel quality-failure events were used to establish biological or operational plausibility and consequence-if-true, not to estimate the frequency or typical severity of routine complaints [36,37,38,39,40]. Spontaneous-reporting systems were treated as signal-generating resources, not denominator-based incidence systems, because under-reporting, incomplete exposure denominators and duplicate reports preclude valid incidence or comparative-risk estimation [41,42,43,44].

Information was abstracted narratively into thematic domains: definitions and boundaries, regulatory and pharmacovigilance foundations, defect–exposure–harm pathways, evidence gaps, signal-management implications, patient-consequence classification, causal-certainty grading and pharmacoepidemiological validation [25,26,27]. No pooled estimates were calculated and no formal risk-of-bias instrument was applied because the evidence corpus combined regulatory texts, guidance documents, outbreak investigations, pharmacovigilance concepts, observational studies and methodological sources that are not directly comparable.

### 2.5. Development of the Proposed Framework

The framework was developed as a distinct methodological process, separate from the evidence synthesis described in Section 2.4. Seed constructs were extracted from quality-system regulation [32], quality risk management [2,45], pharmacovigilance signal management [20], recall evaluation [29], sentinel medicine-quality failures and pharmacoepidemiological reasoning, and are listed in Supplementary S6 Table S7, which records, for each proposed element, the source material from which it derives, the design constraint it satisfies and the alternatives considered and rejected. Supplementary S6 Part A reports the internally reconstructable derivation of the framework elements, the source material informing them, the design constraints, the alternatives considered, the boundary rationales, our general development process and the absence of formal testing, consensus or external review. Development was iterative and author-led and was not documented prospectively using dated versions, independent classification forms or a prespecified development set, so no chronological development record or author-level role allocation can be reported. The proposed minimum data elements and the escalation thresholds are documented in the same way. Fields traceable to an identified regulatory requirement are cited to it in Table 6. The escalation thresholds in Table 7 and Table S6 were generated by the authors from risk factors identified across the reviewed sources; they were not derived from data, were not calibrated against outcomes and have no established performance.

The status of the framework is described in Section 2.7.

### 2.6. Inferential Safeguards

Several safeguards were applied to reduce overinterpretation. First, a complaint was not treated as an adverse reaction unless a clinical event and plausible product-related pathway were described [6,46]. Second, a recall was not treated as evidence of exposure or harm. Third, spontaneous reports were not used to estimate incidence, risk or comparative safety. Fourth, sentinel contamination and toxicity events were used to establish plausibility and consequence-if-true, not to infer the typical risk profile of routine complaints. Fifth, mitigation was treated as risk-management action under uncertainty, not as proof of causality. Causal language was restricted accordingly. These safeguards operate on the evidentiary states defined once, in Section 3, and are not restated elsewhere.

The review uses terms such as “may,” “can,” “plausible,” “candidate patient-relevant complaint,” “patient-relevant triage” and “requires validation” where evidence is incomplete. Stronger causal wording is reserved for events in which defect, exposure pathway, compatible clinical phenotype, chronology and investigation evidence have been established in the cited literature.

### 2.7. Status of the Proposed Framework

The PRCS status, the TRACE sequence, the clinical-consequence classification and the certainty gates constitute a conceptual research model. They are not a patient-safety classification system, a regulatory category, a recall class, a seriousness scale or a validated causality algorithm. They should not be used to classify patients, products or batches in routine practice until the reliability, escalation-validity and decision-utility studies specified in Supplementary S5 have been conducted. Their intended use in the interim is restricted to pilot, quality-improvement and research settings, and to structuring the research questions set out in Section 9.

## 3. Product-Quality Complaints as a Patient-Safety Interface

A product-quality complaint is a report that a medicinal product, package, label, device component, container-closure system or distribution condition may be defective. The report may be true, false, unconfirmed or unresolved after investigation. A suspected quality defect is narrower: it points to possible failure of a product to meet quality requirements, specifications, good manufacturing practice (GMP) or marketing-authorisation expectations. A recall is a regulatory or firm action to remove or correct distributed product; it is not itself evidence that a patient was exposed or harmed.

Patient-safety terminology must remain separate [6,46]. A complaint is a report. A defect is a product state. Exposure is patient or population contact with that state. An outcome is a clinical, operational or psychological consequence after exposure or credible exposure opportunity. A signal is a reason to investigate further. Causal attribution is the judgement that the defect contributed to the outcome. These units are commonly collapsed in operational discussion, especially when a recall notice is the first public document. For patient safety, they should remain separate until evidence links them.

**Figure 1:**
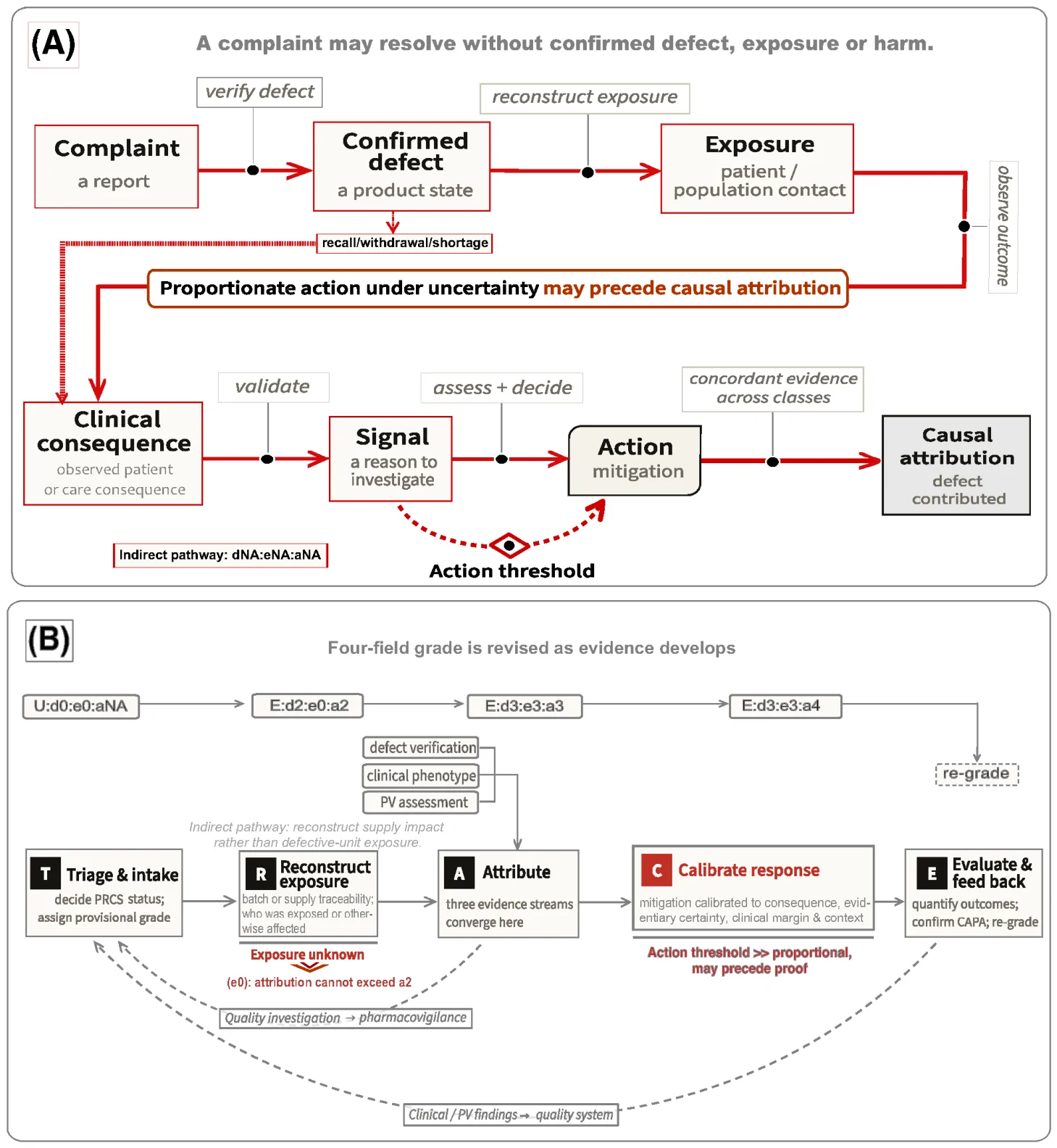
(**A**) Six evidentiary states, plus mitigation as an action that may be taken before the sequence completes, separate a product-quality complaint from a causal attribution: complaint, confirmed defect, exposure, clinical consequence, signal and causal attribution, with mitigation shown off the evidentiary sequence. A complaint may resolve without confirmation of defect, exposure or harm, and proportionate action under uncertainty may precede causal attribution. The diagram is an analytical model, not a mandatory chronological sequence: a clinical event may generate the complaint, exposure may be established before the defect is confirmed, and mitigation may precede several evidentiary steps. (**B**) The proposed TRACE workflow (Triage and intake, Reconstruct exposure, Attribute cautiously, Calibrate response and Evaluate and feed back) carries a revisable grade in which clinical consequence, defect certainty, exposure certainty and outcome attribution are recorded separately. Indirect supply pathways bypass direct defective-unit exposure assessment and take dNA, eNA and aNA. PRCS, patient-relevant complaint status; PV, pharmacovigilance; CAPA, corrective and preventive action.

PRCS is therefore a proposed operational term, not a regulatory category. A PRCS is a routing designation applied to a product-quality complaint when the alleged defect, if confirmed, could plausibly affect patient exposure, sterility, potency, identity, delivery, medication use, treatment continuity or clinical outcome. It does not indicate defect confirmation, exposure confirmation or patient harm, and it does not imply a validated pharmacovigilance signal, an adverse reaction or an incidence estimate. These evidentiary states are shown schematically in Figure 1A.

The designation marks a position in an evidentiary sequence rather than a new phenomenon. Existing frameworks define duties at each end of the interval it occupies: complaint-file and quality-defect obligations govern the product side [31,47], signal-management logic governs the pharmacovigilance side once a candidate concern exists [20], ICH E2E allocates characterised concerns to identified risk, potential risk or missing information [48], recall policy grades hazard once a defect is confirmed [29] and medication-error taxonomies grade severity once an error has occurred [49]; none of the frameworks identified in this review defines the intermediate state. What is not specified in the frameworks reviewed here is the state of a complaint that is neither a confirmed defect nor an adverse-event report but for which patient-level follow-up should begin.

The proposal here is confined to that interval and to three elements within it: separate grading of consequence, scope, defect certainty, exposure certainty and attribution; treatment of missing linkage information as an active evidence state; and specification of the identifiers and study elements required to make the concern evaluable. Table 1 states the relationship between PRCS and formal signal terminology.

## 4. Regulatory and Pharmacovigilance Foundations

Regulatory architecture already treats product quality as a route to possible patient harm rather than as a purely industrial concern. US current good manufacturing practice requires complaint procedures, investigation decisions and records with product and lot detail where known [47]. US field alert reporting requires rapid notification of significant quality problems with distributed products, including bacterial contamination, significant chemical or physical change, labelling or container mix-up, or batch failure, to meet application specifications [30,50]. Recall policy adds a graded health-hazard evaluation, including occurrence of injury, at-risk populations, seriousness, likelihood and immediate or long-range consequences [29]. European and international frameworks point in the same direction [51,52]. EU GMP Chapter 8 requires systems to record, assess, investigate and review complaints and potential quality defects and to test recall capability [31,33]. ICH Q10 embeds complaint handling, deviations, corrective and preventive action (CAPA) and recalls within a pharmaceutical quality system [32]. ICH Q9(R1) makes patient protection the objective of quality risk management and supports proportionate action under uncertainty [2,45]. EMA Good Pharmacovigilance Practices Module IX supplies the pharmacovigilance grammar for signal detection, validation, prioritisation, assessment, recommendation and communication without prejudging causality [20]. Applied in practice, this architecture generates a distribution of post-marketing actions dominated by batch recall, with impurity or stability problems, good-manufacturing-practice non-compliance and labelling or packaging defects among the principal causes [53]; that evidence describes regulatory action and not patient exposure, harm or causality.

For lifecycle pharmacovigilance, complaint-derived concerns should be mapped explicitly to ICH E2E pharmacovigilance-planning logic [48]. Table 2 maps patient-relevant PQC concerns to ICH E2E pharmacovigilance-planning concepts. A batch-confirmed defect with documented harm may become an important identified risk. A plausible defect pathway with incomplete exposure or outcome evidence may be an important potential risk. Poor lot capture, missing exposure windows, weak denominator data or unknown effects in vulnerable groups constitute missing information. Routine pharmacovigilance may be adequate for low-severity, well-characterised complaints; additional pharmacovigilance activities are justified when routine systems cannot reconstruct exposure, assess seriousness or quantify mechanism-matched outcomes. Risk minimisation should be proportionate to consequence-if-true, outcome attribution, clinical margin, supply alternatives and patient dependence on the product. The detailed scenario-level mapping is provided in Supplementary S3 and is an interpretive overlay rather than a formal regulatory classification.

## 5. Evidence Pathways Linking Defect, Exposure and Harm

The evidence linking quality defects to patient outcomes is strongest when the defect mechanism is specific, exposure can be reconstructed and clinical consequences are mechanistically compatible.

### 5.1. Sentinel Events: What They Establish and What They Cannot

Direct toxicity and contamination are the best documented pathways. The New England Compounding Center fungal meningitis outbreak [38], oversulphated chondroitin sulphate contamination of heparin [39,40] and diethylene glycol or ethylene glycol contamination of paediatric medicines [36,37] show that product-quality failures can cause severe, clustered and sometimes fatal outcomes. These events establish that quality failures can cause severe, clustered and fatal outcomes, and they justify proportionate action when the consequence-if-true is high. They establish nothing about the frequency, expected severity or prior probability of harm for a routine complaint, and they are not used here for that purpose. Because such events are salient and heavily documented, they are also the most likely source of overestimation in this field, and the evidence relevant to routine triage is presented separately in Section 5.2. Table 3 summarises mechanism-matched pathways from quality defect to patient outcome and the minimum evidence needed for interpretation.

### 5.2. Routine Complaints and Their Documented Consequences

The categories encountered in routine practice include packaging and labelling deviations, device and delivery-component malfunction, appearance and particulate observations, potency, dissolution and stability concerns, and complaints arising from storage or distribution conditions. No source identified in this review reports the distribution of complaints across these categories. The list is an inventory of the categories that exist and carries no claim about their relative frequency; regulatory alerts and recalls are a selected subset of complaints and cannot be used to infer the composition of the complaint population. The empirical literature on these categories is thinner and is concentrated on the regulatory-action end of the pathway rather than on patient outcomes. A retrospective analysis of safety and quality alerts issued by the Portuguese medicines authority found that quality problems accounted for the large majority of alerts, with batch recall the predominant action and with impurity or stability problems, good-manufacturing-practice non-compliance and labelling or packaging defects among the principal causes [53]. That evidence describes regulatory action and does not establish patient exposure, clinical harm or causality. A retrospective study of medicine-quality complaints recorded in Kenya’s national pharmacovigilance database differentiated reports into suspected quality defects, suspected therapeutic failure and suspected adverse reactions potentially associated with poor-quality medicines, demonstrating that complaints, clinical consequences and pharmacovigilance reports can coexist within one surveillance infrastructure while remaining analytically distinct [55]; that study describes report counts, which are not incidence. A systematic review of interventions addressing the readability, comprehensibility and differentiation of medicine packaging and labels provides the intervention-side evidence for the error-forcing pathway described in Table 3 [54]; that review evaluated interventions and did not establish an effect on downstream patient harm.

Loss of potency, failed dissolution, degradation and cold-chain failure can produce underexposure rather than toxicity. These outcomes are harder to study because they can resemble disease progression, non-adherence, inadequate dose, ordinary treatment failure or therapeutic switching [19]. Latent pathways, such as nitrosamine impurity exposure, require exposure reconstruction and long follow-up [34]. Packaging, labelling, nomenclature and device-function defects can create medication-use failures even when chemical composition is correct [49,56,57].

Biological products, vaccines and advanced therapies concentrate several of these pathways. Thermal or mechanical stress can produce aggregation and subvisible particulates as well as loss of potency, so the mechanism-matched outcome is immunological—hypersensitivity, infusion reaction or anti-drug antibodies with loss of efficacy—and not only underexposure. Delivery-device constituents fail in ways that are clinically indistinguishable from non-response unless the device is retrieved. For this class, batch identification is a legal expectation rather than a matter of good practice [51], yet batch numbers are recorded for only a minority of suspected biopharmaceuticals in the major spontaneous-reporting systems [58], which places the traceability problem described in Section 8 at its most acute precisely where it matters most. For advanced therapy medicinal products, chain-of-identity and chain-of-custody failure creates a wrong-patient hazard with no analogue in conventional supply, and an autologous product cannot be replaced, so mitigation options routinely available for other classes are absent.

System harm is a further pathway. A quality defect can trigger recall, shortage, substitution, delayed treatment, repeat dosing, monitoring burden, patient anxiety or workflow disruption [16]. Shortage-associated mortality after norepinephrine supply disruption [35] is not evidence that most recalls cause harm; it shows that quality and supply disruptions can translate into measurable outcomes when the affected medicine is clinically critical [59].

Two categories deserve separate note. Monitoring and replacement burden generate no adverse-event report and no diagnostic code, so they are largely invisible in the data sources used for safety research, although they are tractable in dispensing, laboratory and contact records. Complaints that investigation does not confirm are recorded as administrative closures and are rarely retained as analysable data, yet they constitute the denominator against which the specificity of any escalation rule must be measured. Both are consequences of how these events are recorded rather than of how often they occur, and the evidence-status column of Table 8 should be read accordingly.

## 6. From Complaint to Patient-Relevant Complaint Status

Not all PQCs are expected to warrant formal signal-management assessment; the proposed escalation criteria require empirical validation [20,60]. A complaint becomes a candidate patient-relevant complaint only when one or more features increase the prior probability that a defective unit reached a patient or a quality-related supply disruption altered care. These features are listed once, in Table S6, which is the single operational list of escalation variables; they are not maintained as a separate list here. Complaints recorded within national pharmacovigilance systems can be differentiated on exactly these lines into suspected quality defects, suspected therapeutic failures and suspected adverse reactions [55], although such report counts are not incidence.

The proposed TRACE sequence is intended to provide a structured approach to this triage without implying validation. It is not intended to replace existing quality investigation, CAPA, recall assessment or pharmacovigilance signal management; its proposed role is to address the interface between these systems, by determining when a product-quality complaint contains sufficient evidence to justify patient-exposure or supply-impact reconstruction and clinical safety assessment (Table 4). Triage and intake determine whether the complaint is patient relevant and assign a provisional consequence and certainty grade. Exposure is reconstructed by product, batch, date, distribution, administration and care setting. Attribution is approached cautiously by integrating product investigation, clinical phenotype, chronology, dechallenge or unintentional rechallenge where available, alternative explanations and pharmacovigilance assessment. Response is calibrated by severity, certainty, clinical margin and supply context. Evaluation and feedback then measure outcomes, CAPA, communication performance and reclassification after investigation. The TRACE workflow is shown in Figure 1B.

TRACE is not a root-cause or corrective-and-preventive-action method and is not proposed as an alternative to one. Root-cause investigation and CAPA take the process or product as their object and ask why a failure occurred and how recurrence is prevented. TRACE takes the patients potentially exposed or otherwise affected as its object and asks who was exposed or otherwise affected, what clinical action is warranted now and which evidence must be preserved before it is lost. The two proceed concurrently and exchange findings in both directions (Figure 1B). A corrective action can be closed while no potentially exposed patient has been identified, and a patient-safety triage can be completed while root cause remains unresolved; neither substitutes for the other. Table 4 provides the comparison. No claim of superiority over existing quality-investigation methods is made, and none would be testable, because the two workflows do not share an endpoint. TRACE precedes formal signal management and feeds it: concerns that pass the documentation, plausibility and priority thresholds enter the detection and validation phases of signal-management logic [20], and those that do not remain within routine quality handling.

### Over-Detection, Specificity and the Base-Rate Problem

The argument that complaints are under-recognised as a patient-safety input has a counterpart that must be stated explicitly: a triage rule that raises sensitivity without attending to specificity will increase escalation. Three sources of false-positive escalation should be distinguished, because they require different responses.

First, complaints that are accurate but not product-related. Administration and use error, storage and handling deviation after dispensing, and product obtained outside the regulated supply chain can each produce a real observed defect whose origin is not the licensed batch [13,61,62].

Second, complaints that are ascertainment artefacts. Recall or media publicity increases reporting of pre-existing observations and shifts attribution, so an apparent cluster may reflect a change in ascertainment rather than a change in the product; the same mechanism is long described for spontaneous adverse-event reporting [41,42,43,44], and the case for filtering incidental events is made in [60].

Third, complaints in which the clinical event has an alternative explanation. Coincidental disease progression, non-adherence, expectation and attribution effects after a patient learns of a recall, and lack of efficacy attributable to the disease rather than to the product all compete with a defect-based explanation. The last is the hardest to exclude, because loss of potency and ordinary treatment failure are clinically indistinguishable without a monitorable pharmacodynamic marker (Section 5.2).

Escalation is not costless. Precautionary withdrawal of a clinically critical medicine can produce substitution, delayed treatment and shortage, and shortage has been associated with excess mortality [35]. A triage framework that raised sensitivity while ignoring specificity would move harm rather than reduce it. Positive predictive value will depend on the prevalence of patient-relevant defects among complaints, which is currently unknown: no complaint-level denominator was identified in this review, and the composition of regulatory alerts [53] describes a selected subset of complaints and cannot be used to estimate that prevalence. Validation studies should therefore report performance across a range of relevant prevalence settings and should evaluate both missed cases and unnecessary escalation. The escalation thresholds proposed here are therefore stated as hypotheses to be evaluated against both error types—missed patient-relevant defects and unnecessary escalations—under an explicit and asymmetric loss function, and not against missed cases alone.

## 7. Classification of Clinical Consequence and Evidentiary Certainty

The classification proposed in this section is a conceptual research model (Section 2.7). We propose a six-level patient-consequence classification intended to facilitate more consistent description of PQCs across quality, pharmacovigilance, medication-safety and outcome-research systems, and to make that description testable. It is a proposed patient-safety overlay, not a recall class, seriousness scale, causality scale, regulatory standard or validated prediction tool. Table 5 summarises the proposed clinical-consequence classification and the separately graded defect, exposure and attribution certainty.

The axis records observed clinical outcome only; exposure is recorded exclusively on the exposure gate (e0–e3), so no level of the consequence axis carries exposure information. Levels A and B are findings, not defaults. A requires either adequate follow-up showing no clinical effect and no management change or positive evidence excluding exposure. B requires documented assessment or monitoring and follow-up sufficient to exclude clinical harm. Where ascertainment was absent or incomplete the case takes U, which records that the outcome is unknown. This preserves the framework’s general rule that missing information is an active evidence state rather than a benign one, and it prevents an unobserved outcome being read as an observed absence. Level A denotes no observed clinical effect and no change in management. Level B denotes additional assessment or monitoring undertaken without any change of treatment and without clinical harm. Level C denotes a change in treatment or product management without clinical harm, including replacement, repeat dosing and intercepted error. Level D denotes temporary non-serious clinical harm. Therapeutic failure, treatment interruption and disease destabilisation are mechanisms rather than severities and are not level-defining: each is graded by the outcome actually observed. Sub-therapeutic anticoagulation corrected by monitoring and dose adjustment is graded C or D, symptomatic hyperglycaemia managed without admission is graded D and graft rejection or any outcome meeting standard seriousness criteria is graded E. Where the consequence of a deviation depends on therapeutic index, critical-dose status or the absence of a monitorable pharmacodynamic marker, that dependence is handled as a triage priority amplifier (Table S6), not as a severity level.

Level E denotes serious non-fatal clinical harm meeting standard seriousness criteria [64,65,66]. Level F denotes death. Clustering is not a severity and is recorded on the scope flag, which may be applied at any consequence level: a cluster of monitoring-only events is recorded as B + S(cluster) and an isolated death as F. A multi-batch or cross-border event is recorded on the same flag. This separation prevents a single fatality and a large non-fatal cluster from receiving the same grade, and preserves the epidemiological information that scope carries.

Outcome attribution uses an author-adapted operational mapping of the WHO-UMC causality category labels [63]. The labels and their ordering are taken unchanged and no category is merged, but the mapping attaches defect-specific evidence requirements to each grade, treats rechallenge as uninformative for a batch-specific defect, and links the labels to separate defect and exposure gates that the WHO-UMC system does not contain. It should therefore not be read as the WHO-UMC system applied without modification, and it has not been validated for defect-to-outcome attribution. All six labels are retained—conditional or unclassified (a0C), unassessable or unclassifiable (a0U), unlikely (a1), possible (a2), probable (a3) and certain (a4). The two data-poor categories are kept distinct because they are not the same finding: a0C denotes evidence that is insufficient or contradictory but capable of being supplemented, whereas a0U denotes a report that cannot be assessed at all. Only the first is answerable by targeted reconstruction, and the distinction therefore carries an operational consequence. A separate state, aNA, is recorded where no clinical outcome requires attribution, exposure has been excluded or direct defect–exposure attribution is inapplicable because the consequence arose through an indirect supply pathway; aNA is explicitly not a WHO-UMC category and is not a grade of causality, and it exists so that such cases are not forced onto a causality scale that has no object. Two judgements that the established causality instruments do not address are graded separately: whether the defect exists (d0–d3) and whether the patient received the affected unit (e0–e3). In complaint-derived assessment, rechallenge is unavailable and the same product from an unaffected lot does not constitute one, so the discriminating evidence for defect attribution is analytical—retained-sample testing, lot concordance and distribution records—rather than clinical. The full category mapping, with the minimum evidence required for each grade, is provided in Supplementary S4 (Table S4a) and the decision rules for each gate in Supplementary S6 Part B.

**Figure 2:**
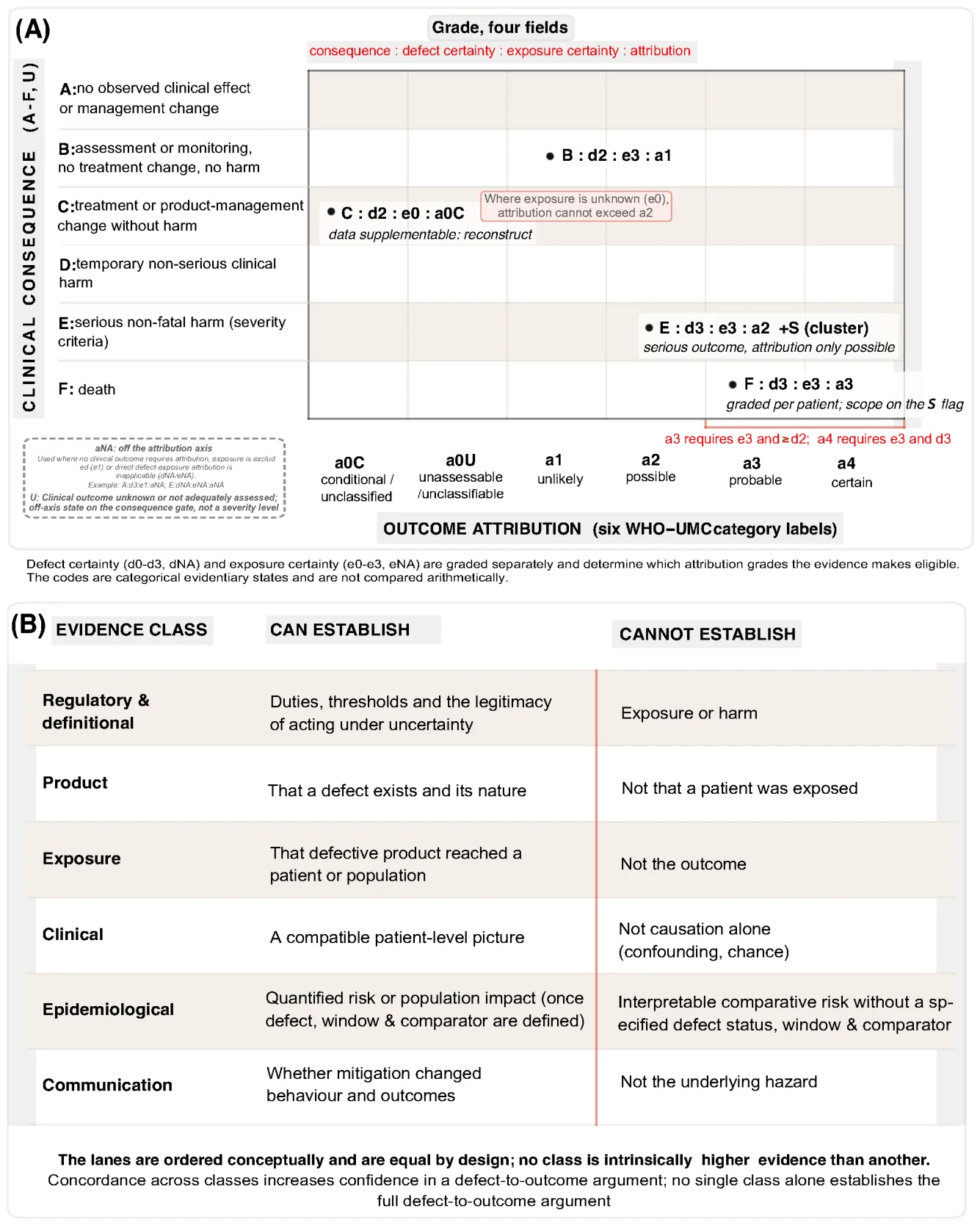
Clinical consequence and outcome attribution as independent dimensions. (**A**) Clinical consequence (A–F, U) and outcome attribution (aNA, a0C, a0U and a1–a4) are represented on separate axes rather than combined into a single score. This permits proportionate action under uncertainty. For example, serious harm within a cluster may warrant action despite only possible attribution (E:d3:e3:a2), whereas strong defect evidence may coexist with a benign clinical state, such as a confirmed defect with exposure excluded (A:d3:e1:aNA). Defect certainty (dNA, d0–d3) and exposure certainty (eNA, e0–e3) are graded independently and determine which attribution grades are evidentially eligible (Section 7). (**B**) Six complementary evidence classes—regulatory and definitional, product, exposure, clinical, epidemiological and communication—address distinct questions. Their order is conceptual and the lanes are intentionally equal; no class is intrinsically superior to another.

The three gates are not independent; their relationship is one of evidence eligibility rather than arithmetic. The codes are categorical evidentiary states, not numerical quantities. Applicable grades may reflect increasing evidential support, but NA and data-deficiency states are not ordered and no codes are compared arithmetically. Four rules apply. An attribution of certain (a4) requires a confirmed defect (d3) and documented exposure (e3). An attribution of probable (a3) requires documented exposure (e3) and at least a strongly suspected defect (d2). Where exposure status is unknown (e0), attribution may rise no higher than possible (a2), because the exposure element of any causal argument is absent rather than negative. Where exposure has been excluded (e1), or where no clinical outcome requires attribution, the case is recorded as aNA rather than as a low causality grade. These rules replace the numeric “lower-of” formulation used in the submitted version, which was internally inconsistent: read arithmetically, e0 would have forced attribution to a0 rather than to the a2 ceiling the same text specified. Severity and certainty must not be compressed into a single score. A grade of E:d3:e3:a2 + S(cluster) denotes serious harm in a cluster, with the defect confirmed and exposure documented, but attribution still only possible. A recalled batch shown not to have reached patients is A:d3:e1:aNA—the defect is confirmed, exposure is excluded and attribution does not arise, which is why the case takes the aNA state rather than a low causality grade. The same defect in a batch dispensed to an identified patient who was then lost to follow-up is U:d3:e3:aNA—defect and exposure both established, outcome unknown. The two cases differ on one gate at a time, which a single composite score could not express. Figure 2 shows clinical consequence and outcome attribution as independent axes.

## 8. Lifecycle Risk Management and Global Signal Transmission

The same defect can have different consequences across regulatory systems. High-capacity systems may detect a quality signal, quarantine lots, notify pharmacies, test retained samples and launch targeted pharmacoepidemiology. Lower-capacity systems may lack batch traceability, laboratory capacity, recall execution, replacement supply or pharmacovigilance reporting [61,62,67]. The global patient-safety question is therefore not only whether the product is defective, but what action minimises net harm for exposed and dependent patients.

Applicability across regulatory contexts is a proposition rather than a demonstrated property of the framework. Its regulatory foundation is drawn predominantly from the United States, the European Union and international harmonised instruments, and no source identified in this review reports the application of comparable triage logic in a setting with limited batch traceability, informal distribution channels or restricted laboratory capacity. Adaptation would require at least the following: a defined set of minimum data elements that does not presuppose electronic dispensing records; substitution of product retention and photographic capture for serialisation where the latter is unavailable; explicit provision for escalation on temporal and setting clustering when lot identification is impossible, as set out in Table S6; a route for cross-border notification where distribution is international [61]; and a net-harm assessment before any withdrawal of an essential medicine for which no substitute is accessible. Whether the framework is usable under those conditions is an empirical question and is included in the validation agenda.

Missing information is not merely an administrative limitation. In complaint-derived safety assessment, missing lot number, unclear exposure date, weak denominator data, incomplete clinical follow-up and poor linkage between quality and clinical systems are often the reason a potentially important safety question cannot be answered. In this setting, missing information should be treated as an evidence state requiring targeted reconstruction, not as a neutral absence of concern.

**Figure 3:**
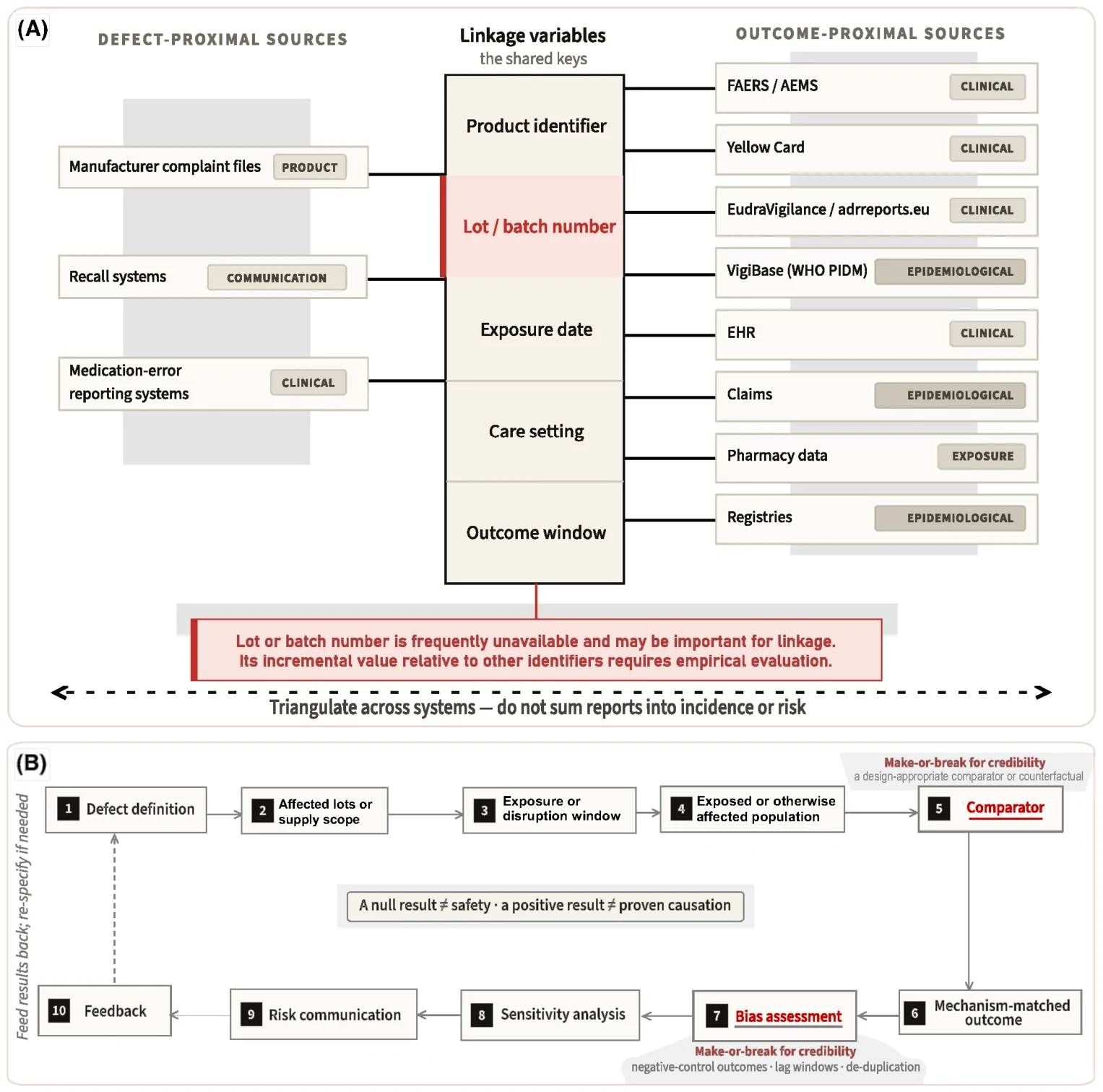
Linked surveillance architecture and evaluable pharmacoepidemiology. (**A**) Fragmented defect-proximal sources—manufacturer complaint files, recall systems and medication-error reporting systems—and outcome-proximal sources—spontaneous-report systems such as FAERS, Yellow Card, EudraVigilance and VigiBase; electronic health records; claims; pharmacy data; and registries—are linked by shared identifiers: product identifier, lot or batch number, exposure date, care setting and outcome window. Batch-number completeness was limited in the FAERS and EudraVigilance study cited [58]; the relative importance of the lot or batch number compared with the other identifiers was not established by any source identified in this review and should be evaluated empirically across data systems. Sources should be triangulated; reports must not be summed into incidence or risk. (**B**) Translating a candidate signal into an evaluable pharmacoepidemiological question requires ten specified elements: defect definition, affected lots, exposure window, exposed population, comparator, mechanism-matched outcome, bias assessment, sensitivity analysis, risk communication and feedback. A null result does not prove safety, and a positive result does not prove causation. AEMS, FDA Adverse Event Monitoring System; FAERS, FDA Adverse Event Reporting System.

Defect-proximal and outcome-proximal data systems are structurally fragmented and are reconciled through shared identifiers, of which the lot or batch number is one; its relative importance compared with other identifiers was not established by any source identified in this review and should be evaluated empirically across data systems. Batch numbers were recorded for only a minority of suspected biopharmaceuticals in FAERS and EudraVigilance and less frequently still for small-molecule products [58], a figure that describes recording completeness at the time studied and may have changed since; the linked surveillance architecture and the elements required to convert a candidate signal into an evaluable pharmacoepidemiological question are shown in Figure 3. We propose that only complaint-derived concerns passing documentation, plausibility and priority thresholds be routed into formal signal-management logic, and that prospective implementation studies determine whether such routing improves detection without excessive escalation. The process should detect credible patterns [41,42,43,44]; validate documentation and plausibility; prioritise by seriousness, preventability, population vulnerability, exposure scope and clinical margin; assess the defect-to-outcome pathway; and communicate what is known, what remains uncertain, what patients and clinicians should do and how to report [68]. Detection, validation, prioritisation, assessment and communication do not establish causality by themselves; they provide a disciplined pathway for action under uncertainty. The WHO-UMC to PRCS mapping is provided in Supplementary S4.

## 9. Pharmacoepidemiological Validation Agenda

Studies best able to support causal interpretation would start with a defect definition from quality investigation, define affected lots and exposure windows from distribution records, identify exposed patients through dispensing or administration data and compare mechanism-matched outcomes against suitable controls. Spontaneous-report systems—such as the FDA Adverse Event Reporting System (FAERS; in 2026 consolidated, with other legacy FDA systems, into the FDA Adverse Event Monitoring System, AEMS [69]), the MHRA Yellow Card scheme, EudraVigilance and the WHO global database VigiBase—can raise candidate signals but cannot, on their own, establish exposure, denominator or incidence [14,70,71,72]. Disproportionality methods apply once a complaint-associated adverse event enters a spontaneous-reporting system; they are not applied to complaint files themselves, which lack the report structure these methods assume.

The operating characteristics of complaint-based triage—sensitivity, specificity, positive and negative predictive value and false-escalation rate—cannot be estimated from current data. Complaint files carry no exposure denominator, patient outcome is not systematically ascertained after a complaint is closed and the target condition is not routinely adjudicated, so no reference standard exists. These quantities are therefore specified as endpoints of the escalation-validity studies in Supplementary S5 rather than reported here. Because predictive value depends jointly on the rule and on the prevalence of patient-relevant defects among complaints, such studies should report prevalence-stratified predictive values, calibration and net benefit under an explicitly asymmetric loss function, rather than a single pooled estimate. Retrospective analyses of quality-related complaints already held in national pharmacovigilance databases [55] and of batch-number completeness in spontaneous-report systems [58] indicate that the data needed for these studies are partly available and partly have to be created.

The outcome must match the mechanism. Infection is relevant for sterility failure, disease destabilisation for underdosing, bleeding or thrombosis for anticoagulant strength errors, glycaemic events for insulin delivery failure, medication errors for labelling or device defects and delays or substitutions for supply-disrupting recalls. Generic “harm” is too imprecise to support causal interpretation.

Validation of the proposed PRCS status; the TRACE workflow; the A–F, U clinical-consequence classification; and the defect, exposure and attribution certainty gates should proceed in a staged fashion. The key domains are classification reliability, escalation validity, lot or batch traceability, clinical usefulness, usability, and burden and public-health responsiveness. Reliability studies should test whether different reviewers classify the same complaint similarly to PRCS or non-PRCS and whether they assign similar consequence and certainty grades. Escalation-validity studies should examine whether PRCS triage identifies cases that later require signal-management action, regulatory communication, recall-linked action or CAPA.

Traceability studies should assess whether structured intake improves lot capture, exposure-window definition and product-identifier completeness. Clinical-usefulness studies should test whether the framework improves follow-up of potentially exposed patients. Usability studies should assess whether pharmacists, quality specialists, pharmacovigilance staff and clinicians can apply the framework consistently without excessive burden. Public-health responsiveness studies should examine whether the framework supports timely mitigation without unnecessary disruption, alarm or shortage-related harm.

The detailed validation agenda is provided in Supplementary S5. Observational validation studies should follow STROBE and, where routinely collected health data are used, RECORD. Studies linking complaint, dispensing and clinical systems should undergo ethics and governance review appropriate to the data and jurisdiction. Where validation is conducted as internal quality-improvement work rather than human-subject research, that status should be formally determined rather than assumed.

## 10. Implementation Implications for Regulators, Manufacturers, Health Systems and Clinical Practice

This section distinguishes three registers, and the distinction is marked throughout. Requirements are actions mandated by an identified instrument and are cited to it. Good practice comprises actions supported by existing but not mandated guidance. Proposals are recommendations made by us for prospective evaluation and are marked as such; none has been validated, and none should be read as guidance.

In pilot, quality-improvement or research settings, clinicians and pharmacists could evaluate whether documenting eligible PQCs using the proposed PRCS structure improves traceability and patient follow-up, particularly where the reported defect could affect exposure, dosing, identity, sterility, potency, treatment continuity, medication use or a vulnerable patient group. This is a proposal for evaluation and is not an existing professional obligation.

The proposed minimum data elements comprise product name, active ingredient, strength, formulation, manufacturer, lot or batch number, expiry date, serialisation or device identifier, storage history, photographs where appropriate, exposure date, route, dose, clinical chronology, seriousness criteria, management change and whether the remaining product was retained. Table 6 proposes minimum data elements for triaging patient-relevant PQCs.

### Patient-Reported Complaints

For defects that become apparent only at the point of use, the patient or carer may be the sole observer, so some patient-relevant defects can be reported by no one else. How often this occurs is not reported by any source identified in this review. It is a premise of the proposed workflow—not an established finding—that patient-originated reports differ in content from professional reports, being relatively richer in terms of use context, timing, storage and product appearance and relatively sparser in terms of batch identity and technical characterisation. No source identified in this review quantifies either the share of patient-relevant complaints that originate with patients or the size of any such content difference, and both are stated here as questions for the validation agenda rather than as descriptions of current practice. Two actions available only to the patient determine whether a complaint remains evaluable: retaining the product and its packaging, and photographing the carton and label before disposal or return. Direct patient reporting routes exist [71], but their intake structures are oriented towards suspected adverse reactions rather than suspected product defects, so a patient reporting a visibly abnormal product without a clinical event may have no natural route. Communication in the other direction must be proportionate: a complaint under investigation is not evidence of harm, and communicating it as though it were can generate the attribution and ascertainment effects described in Section Over-Detection, Specificity and the Base-Rate Problem [68]. Patients and carers are included in the usability domain of the validation agenda, since face validity for a patient-facing intake instrument cannot be established by professional raters alone (Supplementary S5).

We propose that manufacturers and regulators evaluate whether complaint-to-outcome linkage can be made easier. We propose evaluating whether the use of consistent identifiers and clinically meaningful hazard statements in recall notices and field alerts improves complaint-to-outcome linkage. Pharmacovigilance forms should prompt for lot numbers when product quality is suspected, since batch capture in existing spontaneous-report systems is incomplete [58]. Medication-error systems should flag packaging, labelling, nomenclature and device-function failures that may require quality investigation [49,56]; readability, comprehensibility and differentiation of packaging and labels are modifiable determinants of such failures [54], although the effect of intervening on them on downstream patient harm is not established. None of these steps assumes harm; they make harm assessable.

For health systems, the operational priority is preservation of evidence before it disappears. Lot number, packaging, device component, photographs, retained sample, exposure date and clinical chronology are often more valuable than retrospective narrative detail. Once these elements are lost, the complaint may remain administratively closed but scientifically unevaluable. Table 7 proposes frontline escalation thresholds for suspected patient-relevant PQCs.

## 11. Strengths and Limitations

This review integrates quality-system regulation, pharmacovigilance signal management, medication-error prevention, sentinel quality-failure events, recall evidence and pharmacoepidemiological reasoning into a single patient-safety frame. Its central claim is deliberately narrow: PQCs are not proof of patient harm. Some may become candidate patient-safety concerns warranting regulatory or clinical assessment when evidence concerning defect, exposure, mechanism, chronology, batch traceability and outcomes converges.

The contribution rests on three elements that are stated upon first introduction in Section 3 rather than repeated here: separate grading of consequence, scope, defect certainty, exposure certainty and attribution; treatment of missing linkage information as an active evidence state rather than a neutral absence of concern; and specification of the identifiers and the ten evaluable-question elements needed to bridge defect-proximal and outcome-proximal data. These elements are individually present in existing quality and pharmacovigilance frameworks; their integration into a single, batch-aware triage logic is what this review proposes.

The main limitation is that this is a structured narrative synthesis rather than a systematic review or quantitative analysis. Source selection was not protocol-driven and is subject to author judgement. The proposed PRCS framework, TRACE workflow and certainty grading are unvalidated and should be treated as hypotheses for operational testing; their clinical decision utility in particular is unproven and is the first target of the validation agenda. The evidence is strongest for catastrophic contamination, toxic exposure clusters, shortage-associated harm and regulatory architecture. It is weakest for routine complaints causing transient, indirect, latent or patient-burden outcomes. Publication bias, reporting bias, duplicate reports, incomplete denominator data, inconsistent lot capture and heterogeneous coding limit inference.

The framework is presented without any of the evidence that would make it practice ready. Specifically, no empirical dataset was generated or analysed, no validation study was undertaken, inter-rater reliability was not assessed, diagnostic and predictive performance were not evaluated, no usability testing was conducted, no stakeholder-consensus process was held, and there is no evidence that applying the framework improves patient-safety outcomes, lot capture or follow-up. The contribution is therefore conceptual and methodological, and the framework should be treated as a set of hypotheses for prospective evaluation in the sequence set out in Supplementary S5.

The framework was developed by the authors without a formal consensus process and without external review by clinicians, pharmacists, quality professionals, regulators or patients. Face validity and usability are therefore untested, and are the second domain of the validation agenda. Bibliographic searching was confined to PubMed, so eligible sources indexed only in other databases may have been missed. Applicability across regulatory contexts is a proposition rather than a demonstrated property. The review also addresses under-recognition of complaints as a patient-safety input more fully than over-detection; Section Over-Detection, Specificity and the Base-Rate Problem sets out the sources of false-positive escalation, but the review identified no study quantifying their contribution.

The sources added at revision strengthen the regulatory-action and traceability ends of the pathway but do not close the gap between complaint and patient outcome, because the routine-complaint evidence base is dominated by regulatory-action endpoints [53,54,55,58]. That gap remains the principal limitation of the evidence reviewed here.

The pathways by which a quality defect can reach a patient are set out in Table 8. The taxonomy is mechanistic and deliberately non-ordinal: the rows are not ranked by frequency, severity, evidentiary strength or study volume, and no row is presented as better or worse evidenced than another. The final column states, for each pathway, what kind of source was identified during source identification and, where none was found, says so. This replaces the evidence-density figure of the submitted version, which ordered categories from sparse to dense without a counting rule, an appraisal step or an independent check, and therefore asserted a hierarchy the review could not support. Where the final column records that no study was identified, that is a statement about the sources retrieved by this review and about how these events are recorded; it is not a statement that the pathway is rare or that the risk is low.

## 12. Conclusions

PQCs should be neither dismissed as purely manufacturing events nor treated as evidence of patient harm. Whatever safety value they carry is unlikely to be realised without disciplined linkage across the elements set out in Figure 3B; whether such linkage improves patient-safety outcomes has not been demonstrated and is the subject of the validation agenda.

A PRCS framework may help make potentially patient-relevant quality defects more visible to pharmacovigilance and clinical safety systems while preserving uncertainty; this potential benefit requires validation, and the studies that would establish it are specified in Supplementary S5. The next step is validation in real complaint, recall, spontaneous-report, medication-error and clinical-outcome datasets, with attention to inter-rater reliability, lot-number capture, exposure reconstruction, decision utility and whether classification measurably improves patient follow-up and risk minimisation.

## Figures and Tables

**Table 1:** Relationship between the proposed construct and established terminology.

Construct	What It Asserts	What Triggers It	Evidence Required	Who Owns It	What It Does Not Imply
*Product-quality complaint*	That a product, package, label, device component, container-closure system or distribution condition may be defective.	A report from any source.	None beyond the report itself.	Quality unit [31,47].	A defect, patient exposure or harm.
*Suspected quality defect*	Possible failure to meet specifications, GMP or marketing-authorisation expectations.	Assessment of a complaint, deviation or internal finding.	Technical assessment in progress.	Quality unit [31,33].	Confirmation, exposure or harm.
*Confirmed defect*	That the product state departs from specification.	Analytical testing, retained-sample analysis or documented quality-system failure.	Objective product evidence.	Quality unit and competent authority [31,33].	That any patient was exposed.
*PRCS (proposed)*	That the complaint warrants patient-safety triage.	A plausibility test applied to the alleged defect at intake.	The complaint and the plausibility test; no defect confirmation required.	The quality–pharmacovigilance–clinical interface (proposed).	A defect, exposure, a signal, an adverse reaction, an incidence estimate or causality.
*Pharmacovigilance signal (GVP Module IX)*	Information suggesting a new potentially causal association, or a new aspect of a known one, that warrants verifying action.	Detection from one or more sources.	Report-level data sufficient for validation.	Marketing-authorisation holder and competent authority [20].	Established causality.
*Validated signal*	That the concern is documented and plausible enough for assessment.	Signal validation.	Documentation, plausibility and known-versus-new review.	Marketing-authorisation holder and competent authority [20].	Causality or a quantified risk.
*Identified risk, potential risk, missing information (ICH E2E)*	The position of a characterised concern within pharmacovigilance planning.	Safety-specification review.	As specified in ICH E2E.	Marketing-authorisation holder and regulator [48].	That routine pharmacovigilance is by itself insufficient.
*Recall classification (21 CFR Part 7 Subpart C)*	The health hazard presented by a distributed product.	Health-hazard evaluation.	Defect characterisation and distribution data.	Firm and competent authority [29].	That a patient was exposed or harmed.
*Adverse drug reaction*	A noxious and unintended response to a medicinal product.	A clinical event with a plausible product-related pathway.	Clinical documentation [6,46].	Reporter, marketing-authorisation holder, regulator.	That a quality defect was involved.
*Causal attribution*	Direct outcome attribution is the judgement that exposure to the defective unit contributed to the clinical outcome. Mediated attribution through recall, withdrawal or shortage lies outside this gate and is recorded as aNA.	Integration of defect, exposure and clinical evidence.	Analytical confirmation, exposure concordance, compatible phenotype and chronology.	Assessor.	A quantified population risk.

Note: GMP, good manufacturing practice; GVP, Good Pharmacovigilance Practices; ICH, International Council for Harmonisation; PRCS, patient-relevant complaint status. PRCS is a proposed operational status and not a regulatory category.

**Table 2:** Lifecycle pharmacovigilance mapping for patient-relevant product-quality complaints, aligned to ICH E2E pharmacovigilance-planning concepts.

ICH E2E Element	Application to PQC-Derived Concern	Actionable Implication
Important identified risk	Batch-confirmed defect with compatible documented harm.	Include in safety assessment and risk minimisation.
Important potential risk	Plausible defect-to-harm pathway with incomplete exposure or outcome evidence.	Validate signal and define additional data needs.
Missing information	Unknown exposed population, lot capture, denominator, vulnerable group, or long-term outcome.	Specify targeted data collection.
At-risk populations	Children, intensive-care patients, oncology patients, biologic or vaccine recipients, narrow-therapeutic-index therapy users, low-resource settings.	Prioritise follow-up and communication.
Additional pharmacovigilance	Routine reporting cannot reconstruct exposure or outcomes.	Use batch-aware linkage, active follow-up, targeted EHR or claims studies.
Risk minimisation	Credible high-consequence defect or limited clinical margin.	Quarantine, recall, replacement, monitoring, or patient communication.

ICH, International Council for Harmonisation; PQC, product-quality complaint; EHR, electronic health record. ICH E2E mapping is an interpretive overlay and does not denote a confirmed risk classification.

**Table 3:** Mechanism-matched evidence pathways from quality defect to patient outcome.

Defect Pathway	Clinical Consequence to Assess	Minimum Evidence Needed
Sterility or microbial contamination	Infection, inflammatory reaction, outbreak.	Lot concordance, product testing, cultures, exposure timing.
Potency, dissolution, stability or cold-chain failure (loss of active exposure)	Underdosing, disease destabilisation, therapeutic failure.	Affected lots, exposure window, adherence and disease alternatives.
Chemical impurity or contaminant	Acute toxicity or long-latency toxicological concern.	Analytical confirmation, dose reconstruction, follow-up window.
Labelling, packaging, nomenclature, or device failure	Medication error, near miss, wrong dose, delayed treatment.	Use-context reconstruction, product retrieval, human-factor review [54].
Recall or shortage pathway	Substitution, treatment interruption, delayed therapy, system burden.	Distribution scope, dependency, alternatives, comparator period.
Aggregation, subvisible particulates or immunogenicity after thermal or mechanical stress (biological products)	Hypersensitivity or infusion reaction; anti-drug antibodies with loss of efficacy.	Storage and transport excursion records, product appearance and particulate assessment, immunogenicity testing where available, exposure timing.
Container-closure integrity or device-constituent failure (prefilled syringe, pen, autoinjector)	Non-delivery or partial delivery, underdosing, injection-site events.	Device retrieval and testing, dose-accountability reconstruction, use-context review.

**Table 4:** Relationship between the proposed TRACE sequence and established quality, risk-management, recall and signal-management workflows.

Workflow	Object of Assessment	Question Answered	Primary Output	Typical Owner	Relationship to the Others
Root-cause analysis and CAPA (ICH Q10 [32])	Process and product.	Why did the failure occur and how is recurrence prevented?	Corrective and preventive actions with effectiveness check.	Quality unit.	Runs concurrently with TRACE and exchanges findings; does not identify exposed patients.
Quality risk management (ICH Q9(R1) [2,45])	Risk to the patient from product or process.	What is the risk and what action is proportionate?	Risk evaluation and control decision.	Quality unit with cross-functional input.	Supplies the proportionality rule applied at the calibrate-response stage of TRACE.
Recall health-hazard evaluation (21 CFR Part 7 Subpart C [29])	Distributed product.	What hazard does the distributed product present and what recall strategy follows?	Recall classification and strategy.	Firm and competent authority.	TRACE may supply structured patient-level evidence relevant to health-hazard assessment; recall classification and strategy remain governed by the applicable regulatory procedures.
Signal management (GVP Module IX [20])	A candidate safety concern.	Is there a new or insufficiently characterised association warranting action?	Detection, validation, prioritisation, assessment and communication decisions.	Marketing-authorisation holder and competent authority.	TRACE precedes and feeds it; concerns passing the thresholds enter detection and validation.
Proposed TRACE sequence	Patients potentially exposed.	Who was exposed or otherwise affected, what clinical action is warranted now, and what evidence must be preserved?	Triage status, captured linkage variables, clinical follow-up and proportionate mitigation.	The quality–pharmacovigilance–clinical interface (proposed).	Concurrent with quality investigation; precedes signal management; replaces none of them.

Note: CAPA, corrective and preventive action; GVP, Good Pharmacovigilance Practices; ICH, International Council for Harmonisation. TRACE is proposed and unvalidated; the comparison is conceptual and no claim of superiority is made.

**Table 5:** Proposed clinical-consequence classification and separately graded certainty.

Field	Codes	Definition
Clinical consequence	A–F, U	A = no observed clinical effect and no management change; B = additional assessment or monitoring without treatment change or clinical harm; C = treatment or product-management change without clinical harm, including replacement, repeat dosing and intercepted error; D = temporary non-serious clinical harm; E = serious non-fatal clinical harm meeting standard seriousness criteria; F = death. Follow-up condition: A may be assigned when adequate follow-up shows no clinical effect and no management change, or when exposure is excluded by positive evidence (e1). B may be assigned only when additional assessment or monitoring occurred and follow-up was sufficient to exclude clinical harm. Where outcome ascertainment is insufficient to assign any A–F level, record U.
Defect certainty	d0–d3, dNA	Proposed defect-certainty grades, from an existing but uninvestigated or insufficiently characterised defect object to a confirmed defect. d0 = a defect object exists, but it was not investigated or the available evidence is insufficient to classify it; d1 = investigated and defect not confirmed; d2 = defect suspected on partial evidence; d3 = defect confirmed by analytical testing, retained-sample analysis or documented quality-system failure. dNA = direct defect assessment is not applicable because the consequence arises through an indirect supply pathway; any upstream quality defect is documented separately.
Exposure certainty	e0–e3, eNA	e0 = exposure status unknown; e1 = exposure excluded by positive evidence; e2 = exposure possible; e3 = exposure documented with lot concordance. eNA = exposure to a defective unit is not the operative pathway because the consequence arises indirectly through recall, withdrawal or shortage.
Outcome attribution	aNA, a0C, a0U, a1–a4	An author-adapted operational mapping of the six WHO-UMC causality category labels [63], retained without merging: a0C = conditional or unclassified; a0U = unassessable or unclassifiable; a1 = unlikely; a2 = possible; a3 = probable; a4 = certain. Where exposure has been excluded (e1), no clinical outcome requires attribution, or the direct defect–exposure pathway is inapplicable (dNA/eNA), the case is recorded as aNA rather than as a causality grade.
Qualifier flags	S, P, X	S = one or more scope descriptors: isolated, cluster, multi-batch and/or cross-border; P = healthcare-process burden; X = patient-experience burden. Recorded independently; not severity levels.

Note: The grade is a proposed, unvalidated patient-safety overlay; it is not a recall classification, regulatory seriousness scale, or validated causality algorithm. The qualifier flags are nominal: they are neither summed nor ordered, and they are not severity levels. The four core fields are recorded together, with qualifier flags recorded separately; neither the fields nor the flags are combined arithmetically; the evidence-eligibility rules governing which attribution grades the defect and exposure evidence can support are given below and in Supplementary S6 Part B.

**Table 6:** Proposed minimum data elements for triaging a patient-relevant product-quality complaint (for evaluation).

**Data Domain**	**Fields to Capture**	**Provenance**
Report metadata	Reporter type, date, channel, country, care setting.	Author proposal (operational metadata).
Product identity	Product name, active ingredient, strength, formulation, manufacturer, national product identifier.	Requirement: complaint-record content [47].
Batch traceability	Lot or batch number, expiry date, serialisation or device identifier.	Requirement: lot detail where known [47]; field-alert screening [30,50].
Defect characterisation	Defect type, patient-facing and technical description, photographs, retained sample, storage and handling history.	Requirement: nature of complaint and investigation record [47]. Photographs and retained-sample flag: author proposals.
Exposure	Administered or used, route, dose, timing, number of doses, exposure window.	Author proposal; the exposure window is not required by [47].
Clinical detail	Event type, seriousness criteria, vulnerable-patient status, indication, concomitant medicines, alternative explanations.	Good practice: seriousness criteria and alternative explanations [6,46]. Vulnerable-patient status: author proposal.
Management and outcome	Replacement, monitoring, repeat dosing, therapy interruption, medical outcome, communication and reporting routes; escalation rationale, recording why escalation did or did not occur.	Requirement: reply and investigation outcome [47]. Escalation-rationale field: author proposal.

Note: Presented as a structured list of data elements; fields are minimum proposed elements, not a validated case-report form.

**Table 7:** Frontline escalation thresholds for suspected patient-relevant product-quality complaints.

**Escalation Action**	**Provenance**
Retain suspect product and packaging when a high-risk product is involved or patient exposure may have occurred.	Good practice; supports complaint investigation and defect confirmation [47].
Escalate urgently when the suspected defect affects sterility, identity, potency, or delivery of a clinically critical medicine.	Requirement on the NDA or ANDA applicant where field-alert criteria are met; not a duty of frontline clinical or pharmacy staff [30,50]; the urgency threshold is an author proposal.
Capture the lot number before the product is discarded or returned.	Requirement: complaint records include lot detail where known [47].
Seek clinical review when credible exposure matches a serious plausible mechanism.	Author proposal for prospective evaluation.
Route the report to quality assurance, pharmacovigilance, medication safety, and regulatory channels according to mechanism and seriousness.	Requirement on defined actors (manufacturer, applicant or marketing-authorisation holder) [30,31,50]; the routing thresholds are author proposals.

Note: Operational thresholds for frontline staff; they support proportionate action and do not assume that harm has occurred.

**Table 8:** Mechanistic taxonomy of pathways from quality defect to patient consequence (non-ordinal).

Pathway (Non-Ordinal)	Defect Mechanism	Route to Patient Consequence	Evidence Status in the Sources Identified
Altered chemistry	Contamination, impurity, degradation, wrong active ingredient.	Direct toxicity, or exposure to an unintended substance.	Sentinel events and one nationwide cohort of impurity exposure [34,36,37,38,39,40].
Altered sterility	Microbial contamination of a sterile product.	Infection at the site of administration or systemically.	Sentinel outbreak investigation [38].
Altered amount delivered	Loss of potency, failed dissolution, cold-chain excursion, superpotency.	Under- or over-exposure; therapeutic failure or dose-related toxicity, graded by the outcome observed.	Indirect, through substitution and biochemical monitoring endpoints [19].
Altered immunogenicity (biological products)	Aggregation and subvisible particulates after thermal or mechanical stress.	Hypersensitivity, infusion reaction, anti-drug antibodies with loss of efficacy not recoverable by dose adjustment.	Mechanistic and traceability literature; no complaint-anchored outcome study identified [51,58].
Failure of delivery	Container-closure or device-constituent failure in a syringe, pen or autoinjector.	Non-delivery or partial delivery; clinically indistinguishable from non-response without device retrieval.	No complaint-anchored outcome study identified.
Error-forcing presentation	Labelling, packaging, nomenclature or presentation defect with correct chemistry.	Medication error at prescribing, dispensing or administration.	Intervention-side systematic review; error taxonomies [49,54,56,57].
Loss of chain of identity	Chain-of-identity or chain-of-custody failure in an advanced therapy medicinal product.	Wrong-patient administration; no substitute product available.	No study identified; included on mechanistic grounds.
Indirect supply pathway	Any defect severe enough to trigger recall or withdrawal.	Substitution, treatment interruption, delay, shortage; harm arises without any patient being exposed to the defect.	Shortage-mortality study and recall series [16,35,53].
Care-process and experience pathway	Any defect requiring replacement, repeat dosing, additional monitoring or explanation.	Replacement, repeat dosing or additional monitoring is graded on the consequence axis as B or C, as applicable; additional healthcare-process and patient-experience burden is recorded independently using the P and X qualifier flags.	Not routinely recorded as an outcome; visible only in operational data.

Note: Mechanistic taxonomy of pathways from quality defect to patient consequence. Rows are unordered and are not ranked by frequency, severity or evidentiary strength. Absence of an identified study indicates a gap in the retrieved literature and in routine recording practice, and is not evidence of absence of harm. Reproducible evidence map of this field, with a prespecified counting rule and independent appraisal, would be a worthwhile separate study.

## Data Availability

No new empirical dataset was generated or analysed for this article. The review is based on published literature and publicly available regulatory, pharmacovigilance and public-health sources.

## Supplementary Materials

The following supporting information can be downloaded at: https://www.mdpi.com/article/10.3390/healthcare14162597/s1. Supplementary Package: Supplementary S1: Narrative-review search transparency; Supplementary S2: Regulatory mapping of complaint-derived patient-safety actions; Supplementary S3: ICH E2E mapping of complaint-derived safety concerns; Supplementary S4: WHO-UMC signal-management and causality mapping to the PRCS workflow; Supplementary S5: Validation agenda for the proposed PRCS framework; and Supplementary S6: Framework development audit trail, decision rules for the certainty gates and illustrative retrospective applications. Supplementary S1 additionally contains Table S1a, search transparency; Supplementary S4 contains Table S4a, mapping of the WHO-UMC causality categories to the outcome-attribution gate; and Supplementary S6 contains Table S7, the framework-development audit trail, and Table S8, the illustrative retrospective application to published cases [34,35,36,37,38,39,40].

## Abbreviations

The following abbreviations are used in this manuscript:

A–F, U	Proposed clinical-consequence levels, recording observed clinical outcome only, from no observed effect to death. U see below
AEMS	FDA Adverse Event Monitoring System
aNA, a0C, a0U, a1–a4	Outcome-attribution grades. a0C to a4 are the six WHO-UMC causality categories, retained without merging; aNA is a separate operational state used where no clinical outcome requires attribution or exposure is excluded, and is not a WHO-UMC category
CAPA	Corrective and preventive action
d0–d3	Proposed defect-certainty grades, from not investigated to confirmed by analytical testing
e0–e3	Proposed exposure-certainty grades, from exposure status unknown to exposure documented with lot concordance
E2E	Pharmacovigilance planning guideline of the International Council for Harmonisation
EHR	Electronic health record
EMA	European Medicines Agency
EU	European Union
FAERS	FDA Adverse Event Reporting System
FDA	US Food and Drug Administration
GMP	Good manufacturing practice
ICH	International Council for Harmonisation
ICH Q9(R1)	Revised ICH guideline on quality risk management
ICH Q10	ICH guideline on pharmaceutical quality systems
MHRA	Medicines and Healthcare products Regulatory Agency
PQC	Product-quality complaint (plural PQCs)
PRCS	Patient-relevant complaint status
PV	Pharmacovigilance
RECORD	Reporting of Studies Conducted Using Observational Routinely Collected Health Data
STROBE	Strengthening the Reporting of Observational Studies in Epidemiology
TRACE	Triage and intake, Reconstruct exposure, Attribute cautiously, Calibrate response, Evaluate and feed back
U	Clinical outcome unknown or not adequately assessed; an off-axis state on the consequence gate, not a severity level
UMC	Uppsala Monitoring Centre
WHO	World Health Organization
