# Supplementary material for: From Product-Quality Complaints to Patient-Safety Triage: A Structured Narrative Review and Proposed PRCS-TRACE Framework
Source: Healthcare (Basel). 2026 Aug 18;14(16):2597. doi: 10.3390/healthcare14162597 (PMC13512497; doi:10.3390/healthcare14162597)
Supplement: Supplementary file 1 [file healthcare-14-02597-s001.zip › healthcare-4429012-supplementary.pdf]

healthcare

MDPI

Supplementary file

From Product Quality Complaints to Patient-Safety Triage: A Structured Narrative Review and Proposed PRCS–TRACE Framework

Sabihe Cenaj [1] and Erand Llanaj [2,3]

[1] Faculty of Medicine, University of Medicine, Tirana, Albania; cenajsabi@gmail.com
[2] Working Group Translational Research and Health Services Research in General Practice, Medical Faculty OWL, Bielefeld University, 33615 Bielefeld, Germany; erand.llanaj@uni-bielefeld.de
[3] Department of General Practice and Family Medicine, Medical Faculty OWL, Bielefeld University, 33615 Bielefeld, Germany

Contents

Supplementary S1. Narrative-review search transparency....2
Table S1. Source-identification domains and evidentiary functions for the structured narrative review 3
Table S1a. Search transparency for the structured narrative review 3
Supplementary S2. Regulatory mapping of complaint-derived patient-safety actions 6
Table S2. Regulatory interfaces linking product-quality complaints to patient-safety action....6
Supplementary S3. ICH E2E mapping of complaint-derived safety concerns....8
Table S3. Mapping patient-relevant complaint statuses to ICH E2E pharmacovigilance-planning categories....8
Supplementary S4. WHO–UMC signal-management and causality mapping to the PRCS workflow 10
Table S4. Translation of WHO–UMC signal-management phases into a PRCS workflow....10
Table S4a. Operational mapping of the WHO–UMC causality category labels to the outcome-attribution gate....10
Supplementary S5. Validation agenda for the proposed PRCS framework 12
Table S5. Validation domains, study designs and candidate metrics for the proposed PRCS framework....13
Table S6. Candidate escalation-threshold variables for PRCS triage....14
Supplementary S6. Framework development, decision rules and illustrative application....14
Table S7. Framework-development audit trail 14
Table S8. Illustrative retrospective application to published cases....14
Sources 15

healthcare

MDPI

Supplementary S1. Narrative-review search transparency

This article was conducted as a structured narrative review designed to clarify concepts, regulatory interfaces and operational research needs at the boundary between product-quality systems and pharmacovigilance. It was not designed as a systematic review, meta-analysis, incidence review or quantitative risk assessment. No formal review protocol was registered and no quantitative synthesis was undertaken.

The source-identification strategy combined bibliographic searching with purposive retrieval of primary regulatory, pharmacovigilance and public-health documents. PubMed was used as the core biomedical database. Targeted retrieval was conducted from official repositories and normative sources, prioritising the US Food and Drug Administration, the electronic Code of Federal Regulations, the European regulatory and GMP framework, the International Council for Harmonisation, the World Health Organization and the Uppsala Monitoring Centre. Citation tracking of seminal regulatory, pharmacovigilance and pharmacoepidemiological sources was used to identify additional materials relevant to the proposed framework. Searches and source retrieval were completed up to 5 June 2026.

The review question was operational rather than effect-estimation focused: when does a product quality complaint become patient relevant, and how should that transition be described, triaged, documented and studied without implying confirmed harm or causality? Sources were selected when they informed one or more of the following domains: complaint handling, quality defects, pharmacovigilance planning, signal detection or signal management, recall or risk-reduction action, medicines-quality surveillance, pharmacoepidemiological validation or patient-safety consequences of quality failures.

The search was intentionally concept driven. The main concept clusters were product quality complaints and quality defects; pharmacovigilance and signal management; recalls, field alerts and regulatory action; substandard, falsified, contaminated or degraded medicines; packaging, labelling, device-function and administration failures; and pharmacoepidemiological or validation methods.

The PubMed search string was:

(“product quality complaint*” OR “quality defect*” OR “medicines quality” OR “substandard medicines” OR “falsified medicines” OR contamination OR impurity OR recall OR “field alert” OR sterility OR potency OR degradation OR “packaging error” OR “label* error” OR “device malfunction”) AND (pharmacovigilance OR “signal management” OR “drug safety” OR pharmacoepidemiology OR “adverse event*” OR “medication error*” OR recall OR shortage OR “patient safety”)

Official-source retrieval prioritised documents with direct normative or surveillance relevance, including complaint-file requirements, field-alert obligations, recall policy and health-hazard evaluation, pharmaceutical quality-system expectations, pharmacovigilance planning, quality risk management, medicines-quality surveillance and official signal-management descriptions. Where multiple versions existed, the most current official version was preferred, while historically foundational documents were retained when they remained necessary for interpretation.

Study selection was purposive and interpretive. Inclusion favoured official regulatory or public-health documents; landmark case reports, outbreaks or contamination events demonstrating plausible defect-to-harm pathways; papers clarifying signal concepts or pharmacoepidemiological validation; and sources directly relevant to the proposed PRCS, TRACE and consequence–certainty framework. Exclusion favoured sources that were duplicative, purely descriptive without conceptual or operational relevance, non-English where no reliable English official version was available or unrelated to medicinal-product quality and patient-safety interfaces.

Because this article is a structured narrative review, no PRISMA 2020 study-selection flow diagram is provided. The source-identification process was targeted and iterative rather than protocol-driven, and the review does not claim exhaustive identification, screening or inclusion of all eligible evidence. Record counts, title-and-abstract screening counts and full-text assessment counts were not prospectively logged during source identification. They are therefore not reconstructed retrospectively as if they arose from a predefined systematic process. The final evidence base should be interpreted as a structured, source-prioritised narrative synthesis designed to support conceptual clarification, regulatory mapping and framework generation. It should not be interpreted as an incidence review, effectiveness review, risk-quantification exercise or quantitative evidence synthesis.

Where several documents addressed the same issue, the selection rule was applied explicitly rather than case by case: the most current official version was preferred, and historically foundational documents were retained only where they remained necessary for interpretation. The strategy was concept driven and purposive, and no procedure specifically targeted contradictory or negative studies; this asymmetry is stated as a limitation in the main text.

Bibliographic searching was confined to PubMed; no second bibliographic database was searched. Eligible sources indexed only in other databases may therefore have been missed. Because the review's function was conceptual and regulatory mapping rather than exhaustive evidence identification, and because the regulatory corpus was retrieved directly from primary repositories rather than through bibliographic indexing, we judged single-database searching acceptable for that function; it would not be acceptable for an incidence, effectiveness or prevalence question, and no such question is addressed here. Table S1a reports the elements required to reproduce the source-identification process.

Table S1. Source-identification domains and evidentiary functions for the structured narrative review

Source category	Platforms / repositories	Core purpose in review	Time window / status
Bibliographic database	PubMed	Biomedical and pharmacoepidemiological literature; sentinel events; validation methods	Through 5 June 2026
US regulatory sources	FDA; eCFR	Complaint files; field alerts; recalls; FAERS/AEMS terminology and surveillance context	Current versions accessed in 2026
International harmonisation	ICH	E2E pharmacovigilance planning; Q10 quality system; Q9(R1) risk management	Current/landmark harmonised texts
European quality framework	EU GMP / EudraLex	Complaints, quality defects, recalls, signal-management context	Current versions cited where available
Global medicines-quality surveillance	WHO	Substandard/falsified medicines burden; GSMS architecture and activity reports	Current fact sheet plus activity reports
Global pharmacovigilance signal sources	UMC	Signal definition, VigiBase context, official signal-management phases	Current official pages accessed in 2026
Citation tracking	Backward/forward citation review	Seminal conceptual and regulatory references	Ongoing through final drafting

Table S1a. Search transparency for the structured narrative review

Element	Methodological detail
**Bibliographic database and search dates**	PubMed was the only bibliographic database searched. Source identification was completed through 5 June 2026. No second bibliographic database was searched.

**Search strategy**	The complete PubMed search string is reproduced verbatim in Supplementary S1.
**Official repositories and regulatory sources consulted**	Supplementary searches of official regulatory, pharmacovigilance and public-health sources included: the US Food and Drug Administration webpages and repositories relating to product complaints, field alert reports, recalls, the FDA Adverse Event Reporting System and the Adverse Event Monitoring System; the Electronic Code of Federal Regulations, including 21 CFR §211.198, 21 CFR §314.81(b)(1) and 21 CFR Part 7, Subpart C; EudraLex Volume 4, Chapter 8; European Medicines Agency scientific-guideline and Good Pharmacovigilance Practices webpages; International Council for Harmonisation guidelines E2E, Q9(R1) and Q10; World Health Organization webpages and Global Surveillance and Monitoring System reports concerning substandard and falsified medical products; and Uppsala Monitoring Centre resources concerning signal detection, signal management and VigiBase.
**Citation tracking and supplementary source identification**	Backward citation tracking was performed from selected regulatory, pharmacovigilance and pharmacoepidemiological sources. Forward citation searches were also undertaken for sources judged to be conceptually or historically important. Citation tracking continued during manuscript development through completion of the final draft. This process was supplementary and was not conducted as a prospectively specified systematic citation search.
**Eligibility criteria**	**Inclusion criteria:** official regulatory, standards-setting or public-health documents; landmark case reports, outbreaks or contamination events demonstrating a plausible pathway from a medicinal-product quality defect to patient harm; sources clarifying pharmacovigilance signal concepts or approaches to pharmacoepidemiological validation; and sources directly informing the proposed conceptual and operational framework. **Exclusion criteria:** duplicate or substantively redundant sources; sources that were exclusively descriptive and did not contribute material conceptual, evidential or operational information; non-English sources for which no reliable official English version was available; and sources not relevant to the interface between medicinal-product quality and patient safety.
**Language restrictions**	Sources were restricted to English-language publications or official English-language versions of documents originally issued in another language.
**Date restrictions and version selection**	No publication-date restriction was applied. For regulatory and technical documents, the most current official version available at the time of source identification was generally preferred. Earlier documents were retained when they were historically foundational or necessary to interpret the development of regulatory or scientific concepts.
**Screening and source-selection process**	Source identification and selection were conducted for a narrative and conceptual review rather than through a prospectively registered systematic-review process. Records were not managed in a formal review database and the numbers identified, screened by title or abstract and assessed in full text were not prospectively recorded. These counts have not been reconstructed retrospectively because doing so could imply a level of procedural documentation that was not maintained contemporaneously.

**Number of reviewers and reviewer roles**	**SC and EL conducted the PubMed searches, searched official repositories, screened candidate sources, made final inclusion decisions and performed thematic or conceptual abstraction.**
**Resolution of disagreements**	Differences concerning source eligibility, interpretation or thematic classification were resolved through discussion between the authors, with reference to the stated eligibility criteria and the underlying source material. No formal third-party adjudication procedure was used. Where consensus could not be reached, the source was not used to support a definitive claim, or the interpretive uncertainty was explicitly retained in the manuscript
**Search for disconfirming or contradictory evidence**	No prospectively specified procedure was used to identify evidence that contradicted the proposed framework, negative studies or examples in which suspected quality defects were not substantiated or were not associated with patient harm. This limitation is reported in the main text because it may have increased the risk of confirmation bias and selective emphasis.

healthcare

MDPI

Supplementary S2. Regulatory mapping of complaint-derived patient-safety actions

This Supplementary maps the proposed patient-relevant complaint status (PRCS) construct to existing quality-system, phar-macovigilance, recall and medicines-quality-surveillance expectations. PRCS is proposed as an operational overl ay, not as a new legal category, regulatory standard or recall class. Its purpose is to identify when a complaint should r emain within routine quality handling, and when it should additionally trigger patient-safety triage, exposure reconstr uction, signal-management logic or regulatory communication.

The regulatory architecture already contains the elements needed for this bridge. Complaint-file requirements preserve traceability; field-alert obligations identify distributed quality problems that may require rapid notification; recall policy introduces health-hazard evaluation and response calibration; ICH Q10 links complaints, deviations, CAPA and lifecycle improvement; ICH Q9(R1) supports risk-based action under uncertainty; EU GMP Chapter 8 frames complaint and quality-defect handling as a patient-protection function; WHO medicines-quality surveillance addresses cross-border public-health implications; and UMC signal-management logic provides a staged approach for detection, validation, prioritisation, assessment and communication.

PRCS does not replace these systems; it connects them. A complaint should remain in routine quality handling when no credible patient-safety pathway is apparent. A complaint should be managed as a PRCS when the alleged defect could plausibly alter product identity, sterility, potency, delivery, medication use, treatment continuity or clinically relevant exposure. In such cases, quality-system actions and patient-safety actions should proceed in parallel and should be explicitly cross-referenced.

Table S2. Regulatory interfaces linking product-quality complaints to patient-safety action

Primary source / framework	Official expectation	Implication for a PRCS workflow	Minimum operational action
21 CFR 211.198 complaint files	Complaint records should include, where known, product name/strength, lot number, complainant, nature of complaint and reply; records should document investigation findings or the reason no investigation was done.	PRCS intake must preserve core traceability fields and record why escalation did or did not occur.	Capture product, lot/batch, defect description, exposure possibility, decision maker and escalation rationale.
21 CFR 314.81(b)(1) and FDA field-alert guidance	NDA/ANDA applicants must report specified significant quality problems in distributed product within three working days; examples include labelling mix-up, contamination, significant deterioration or failure to meet specifications.	A PRCS with apparent distributed-batch significance may require parallel quality investigation and field-alert assessment.	Add an explicit field-alert screening question at intake for distributed product and serious quality failure.
21 CFR Part 7 Subpart C recalls	Recall policy emphasises health-hazard evaluation, recall classification, recall strategy, public warning where needed and continued availability of essential products.	PRCS response calibration should consider patient harm if true, scope of exposure, clinical seriousness and consequences of product withdrawal or shortage.	Add structured hazard assessment and supply-context assessment before recommending recall-linked actions.

Primary source / framework	Official expectation	Implication for a PRCS workflow	Minimum operational action
ICH Q10 pharmaceutical quality system	Complaint, rejection, recall, deviation, inspection findings and trend signals should feed CAPA and continual improvement across the product lifecycle.	PRCS cases should not sit outside the PQS; they should become a route for CAPA, trend review and feedback into product/process improvement.	Link each escalated PRCS to CAPA status, root-cause analysis and effectiveness review.
ICH Q9(R1) quality risk management	Quality risk management applies across development, manufacturing, distribution and lifecycle decision-making.	PRCS triage should be explicitly risk based, especially when evidence is incomplete but patient consequences could be serious.	Grade consequence-if-true, certainty, exposed population and availability of substitutes.
EU GMP Chapter 8	Systems must record, assess, investigate and review complaints and potential quality defects; confirmed defects with recall or abnormal-restriction implications must be notified to competent authorities in a timely manner; risk-reducing actions may be needed before full root-cause resolution.	PRCS logic fits naturally as the patient-facing extension of Chapter 8 quality-defect management.	Require early risk-reduction decision points, authority-notification review and batch-distribution review.
WHO substandard/falsified product framework and GSMS	Detection, reporting and proportionate response should protect patients while avoiding unnecessary shortages; GSMS supports sharing of suspect-product information.	For internationally distributed or cross-border quality events, PRCS handling should consider public-health notification and cross-jurisdiction signal transfer.	Add a global-distribution/cross-border flag and a WHO-alert review question where relevant.
UMC signal-management model	Signal management proceeds through detection, validation, prioritisation, assessment and communication.	Complaint-derived patient-safety concerns can be handled with formal signal logic without presuming causality.	Route candidate PRCSs into a five-phase signal workflow with documented transitions.
FDA adverse-event reporting (FAERS/AEMS)	FAERS and other legacy systems into the FDA Adverse Event Monitoring System (AEMS); FAERS remains the name used across two decades of post-marketing literature and historical data for drug/biologic adverse events, medication errors and quality complaints resulting in adverse events.	Terminology should distinguish the modernised platform from the longstanding database underlying most cited evidence, without implying that spontaneous reports measure incidence.	Use “FAERS” for the legacy database and the cited literature; use “AEMS” for the consolidated platform from 2026 onward.

Supplementary S3. ICH E2E mapping of complaint-derived safety concerns

ICH E2E describes pharmacovigilance planning as a lifecycle activity built around a safety specification and a pharmacovigilance plan, with explicit attention to important identified risks, important potential risks and important missing information. For the purposes of this review, complaint-derived concerns should be translated into this architecture before deciding whether routine pharmacovigilance is sufficient or whether additional activities are justified. This mapping is interpretive and is not a regulatory classification of any specific product.

PRCS is therefore a proposed upstream triage status. It may feed into risk characterisation, signal assessment or additional pharmacovigilance, but it does not itself constitute an identified risk, a potential risk, a validated signal or a causal finding. A confirmed defect with documented exposure and mechanism-matched harm may align with an important identified risk. A credible defect pathway with plausible exposure but incomplete confirmation may align with an important potential risk. A recurrent complaint pattern with weak lot capture, uncertain denominator data or unknown vulnerable-population effects may primarily expose important missing information. An isolated low-severity complaint with no plausible patient-safety pathway should remain in routine quality handling.

Table S3. Mapping patient-relevant complaint statuses to ICH E2E pharmacovigilance-planning categories

PRCS scenario	Best-fit E2E category	Why it fits	Usual evidence threshold	Likely pharmacovigilance response
Confirmed defect with documented patient exposure and mechanism-matched harm	Important identified risk	The defect–outcome association has moved beyond suspicion into a clinically meaningful, risk-bearing relationship	Confirmed defect plus credible exposure and compatible clinical pattern	Routine PV plus targeted evaluation, labelling/risk-communication review, possible recall/risk minimisation
Credible defect pathway with plausible exposure and compatible outcomes, but incomplete confirmation or competing explanations	Important potential risk	The causal pathway is credible but not yet sufficiently characterised	Complaint cluster, plausible mechanism, chronology, partial investigation or early spontaneous reports	Additional PV or focused follow-up, intensified signal monitoring, possible targeted analytic study
Recurrent complaint pattern with weak lot capture, uncertain exposure denominator or unknown vulnerable-population effect	Important missing information	The key problem is not merely uncertainty of causation but missing data needed to interpret risk	Sparse traceability, under-captured batch data, missing subgroup data, unclear follow-up	Methods strengthening, better data capture, registry/claims/EHR linkage, active follow-up
Isolated low-severity complaint with no plausible patient-safety pathway	Not an E2E safety issue requiring escalation at present	Not every complaint is a pharmacovigilance problem	Complaint only, no exposure relevance, no pattern	Routine quality handling only
Complaint associated with medication-error forcing conditions	Potential or identified risk depending on	The risk pathway may be mediated through use error	Error-forcing design plus exposure opportunity ±	Cross-function medication-safety review, error coding, targeted risk

PRCS scenario	Best-fit E2E category	Why it fits	Usual evidence threshold	Likely pharmacovigilance response
from labelling, packaging or device-function error	exposure/harm evidence	rather than chemistry or sterility	harm documentation	communication, possible design CAPA
Recall-triggering quality issue with uncertain patient consequences but high consequence-if-true	Usually important potential risk initially	Precautionary action may be needed before causality is resolved	Distributed product plus serious defect type plus unresolved exposure/outcome certainty	Parallel recall/risk-reduction action and formal signal prioritisation

Routine pharmacovigilance should remain the default for all products. However, ICH E2E makes clear that products with important identified risks, important potential risks or important missing information may require additional actions beyond routine surveillance. Applied to complaint-derived concerns, the decision to escalate should not be binary or rhetorical; the complaint should be translated into an E2E-aligned question about whether the case already represents an identified risk, constitutes a potential risk requiring characterisation, or mainly exposes missing information that prevents reliable risk interpretation.

Supplementary S4. WHO–UMC signal-management and causality mapping to the PRCS workflow

A PRCS should be understood as a candidate signal input, not as a validated signal or established causal finding. The WHO–UMC signal-management model is useful because it separates detection, validation, prioritisation, assessment and communication. This staged logic preserves uncertainty while supporting proportionate action.

Applied to complaint-derived concerns, detection asks whether the report suggests a new or insufficiently characterised defect-to-harm pathway. Validation asks whether the documentation, product identity, lot plausibility, chronology and defect mechanism are credible enough for further assessment. Prioritisation asks how important the issue would be for patients or public health if the concern were true. Assessment integrates quality findings, exposure reconstruction, clinical compatibility, alternative explanations and regulatory context. Communication specifies who needs to know, what is known, what remains uncertain and which practical actions are required.

Global signal context matters because complaint-derived patient-safety concerns may cross national quality systems, especially where products, batches or supply chains are international. In such cases, a PRCS workflow should support structured signal transfer beyond the originating quality system while avoiding inflation of unverified complaints into causal claims.

Table S4. Translation of WHO–UMC signal-management phases into a PRCS workflow

WHO–UMC phase	Operational question for complaint-derived concerns	PRCS workflow translation	Decision output
Detection	Is there information suggesting a new or insufficiently characterised defect-to-harm pathway?	Intake, initial triage, duplicate check, cluster check, mechanism flag, batch/lot flag	Candidate PRCS or routine quality complaint
Validation	Is the concern credible enough for further assessment?	Confirm documentation quality, product identity, lot plausibility, chronology, defect plausibility, known-vs-new review	Validated PRCS for prioritisation or closed/no escalation
Prioritisation	If true, how important is the issue for patients or public health?	Grade seriousness, exposed population, vulnerability, essential-product status, recurrence, cluster scope, shortage implications	High / moderate / low priority
Assessment	What is the most likely explanation, and what further evidence is needed?	Integrate quality findings, exposure reconstruction, clinical compatibility, alternative explanations, literature/regulatory context	Initial judgement: monitor, investigate, communicate or refute
Communication	Who needs to know, what is known and what remains uncertain?	Internal alert, manufacturer/regulator contact, clinician/patient messaging, follow-up request, documentation of uncertainty	Proportionate communication package

Table S4a. Operational mapping of the WHO–UMC causality category labels to the outcome-attribution gate

WHO–UMC causality category	Code used here	Minimum evidence required	Note

Certain	a4	Defect confirmed (d3), exposure documented (e3), compatible clinical phenotype and chronology, alternative explanations excluded.	Rechallenge is not available for a batch-level defect and is not required; the same product from an unaffected lot does not constitute one.
Probable / likely	a3	Defect confirmed or strongly suspected (d2 or d3), exposure documented (e3), compatible chronology, alternative explanations unlikely.	Requires at least d2 and e3 under the evidence-eligibility rules (Supplementary S6 Part B).
Possible	a2	Compatible chronology with a plausible defect pathway; alternative explanations not excluded.	Ceiling grade where exposure status is unknown (e0).
Unlikely	a1	Chronology or mechanism incompatible, or a more probable alternative explanation is available.	Distinct from "not investigated", which is d0 on the defect gate.
Conditional / unclassified	a0C	Data insufficient or contradictory, but capable of being supplemented by further information.	Retained as a distinct code. This is the state that targeted reconstruction can resolve, so it carries an operational consequence that a0U does not.
Unassessable / unclassifiable	a0U	The report cannot be assessed: information is missing or contradictory and cannot be supplemented.	Retained as a distinct code. No reconstruction is possible, so the case is closed on the attribution gate rather than followed up.
(Not a WHO–UMC category)	aNA	No clinical outcome requires attribution, or exposure has been excluded (e1).	A separate operational state, not a causality grade and not part of the WHO–UMC system. It exists so that cases with no object for attribution are not forced onto a causality scale.

This mapping preserves an important distinction. Detection and validation ask whether a complaint is worthy of structured investigation; they do not establish causality. Prioritisation asks how dangerous delay might be if the concern is real. Assessment then integrates quality data, exposure evidence and clinical information. Communication should be proportional, explicit about uncertainty and linked to practical actions such as product quarantine, replacement, enhanced follow-up or regulator/manufacturer notification. Because UMC describes VigiBase as the world's largest source of post-marketing safety data, complaint-derived patient-safety concerns that cross borders may need structured signal transfer beyond the originating quality system.

healthcare MDPI

Supplementary S5. Validation agenda for the proposed PRCS framework

The PRCS construct, the TRACE workflow, the clinical-consequence classification and the separately graded defect, exposure and attribution certainty gates should be treated as hypotheses for operational testing, not as validated regulatory standards. Validation should proceed in staged fashion and should examine whether the framework is reliable, feasible, information preserving, clinically meaningful and decision useful.

The first validation task is classification reliability: whether different trained users classify the same complaint similarly as PRCS or non-PRCS, and whether they assign similar consequence and certainty levels. The second is escalation validity: whether PRCS triage identifies cases that later require signal-management action, regulatory communication, recall-linked action or CAPA. The third is lot and batch traceability: whether structured PRCS intake improves capture of the identifiers needed to reconstruct exposure. The fourth is clinical usefulness: whether the framework improves follow-up of potentially exposed patients and reduces missed patient-safety pathways.

The fifth is usability and burden: whether pharmacists, quality specialists, pharmacovigilance staff and clinicians can apply the framework consistently without excessive workload. The sixth is public-health responsiveness: whether the framework supports timely mitigation without unnecessary disruption, alarm or shortage-related harm.

Reliability analyses should be prespecified according to the data type and number of raters. Chance-corrected agreement statistics should be used for categorical escalation decisions and nominal classification outputs; weighted methods are preferable for ordered consequence levels. Because chance-corrected agreement is prevalence dependent and escalation is expected to be uncommon, a prevalence-robust agreement statistic should be reported alongside weighted kappa, and sample size should be planned for precision on the selected statistic rather than for a significance test. Sample-size planning should reflect the anticipated prevalence of escalated cases, number of raters, number of categories and target precision or power for the selected agreement statistic. Outcome-oriented validation should report discrimination, calibration and decision utility. Time-sensitive implementation outcomes should be analysed with time-to-event or segmented before–after methods where appropriate. If data are clustered by site, product or batch, hierarchical models should be prespecified.

Escalation-validity studies require three further specifications. First, predictive values should be reported by prevalence stratum rather than pooled, because predictive value is a joint property of the rule and of the base rate of patient-relevant defects among complaints. Second, calibration should be reported alongside discrimination, and decision utility expressed as net benefit under an explicitly declared and asymmetric loss function, since a missed exposed-lot cluster and an unnecessary escalation that precipitates a shortage are not equivalent errors. Third, adjudication of the reference outcome should be blinded to the assigned grade, to prevent incorporation bias. Traceability and clinical-usefulness studies should use interrupted time series with prespecified change points in preference to a simple before-and-after comparison, so that the intervention is separated from secular trend and regression to the mean.

Linked exposure-outcome studies should use active-comparator, new-user designs with negative-control outcomes, lag windows and de-duplication, and should handle the interval between dispensing and recall notification so that immortal time is not attributed to the exposed cohort. One primary metric should be prespecified per validation domain, with all others reported as secondary.

The preferred validation datasets are those that allow linkage across quality investigation, product traceability and patient outcomes. Depending on jurisdiction and feasibility, this may include complaint files, retained-sample investigations, batch distribution records, dispensing claims, administration records, electronic health records, recall logs and

spontaneous pharmacovigilance data. FDA's real-world-evidence framework explicitly recognises routinely collected data sources such as electronic health records, claims, registries and observational designs as important for evidence generation, while CIOMS emphasises that signal-detection metrics should align with the operational objectives of the programme. Observational validation studies should follow STROBE, with RECORD extensions where routinely collected health data are used.

Validation studies should undergo ethics and governance review appropriate to the data and jurisdiction. Where identifiable or potentially re-identifiable health data are linked across complaint, dispensing and clinical systems, the legal basis for data sharing, de-identification strategy and access controls should be documented prospectively. If validation is conducted as internal quality-improvement work rather than human-subject research, that status should be formally determined rather than assumed.

Escalation thresholds should remain pragmatic but explicit. Distributed-batch problems, sterility or contamination concerns, wrong-product or wrong-strength concerns, device non-delivery, vulnerable-population exposure, recurrent same-lot complaints, complaint-linked adverse events, treatment interruption, essential-medicine dependence and strong temporal or setting clusters should increase escalation priority. Missing lot number should not automatically prevent escalation when other evidence suggests a credible patient-safety pathway; rather, it should be treated as missing information that limits interpretation and requires targeted reconstruction. Table S6 is the single operational list of escalation variables; the features named in Section 6 of the main text are reconciled with it and are not maintained as a separate list.

Table S5. Validation domains, study designs and candidate metrics for the proposed PRCS framework

Validation domain	Core question	Suggested design	Candidate data sources	Primary metrics
Classification reliability	Do different reviewers classify the same complaint similarly?	Blinded multi-rater study using historical complaint vignettes and live cases	Manufacturer complaint files; hospital incident reports; regulator case summaries	Inter-rater agreement for PRCS yes/no; agreement for consequence level; agreement for certainty level
Escalation validity	Does PRCS triage correctly identify cases later judged to require signal-management or regulatory action?	Retrospective cohort of complaints with known downstream outcomes	Complaint files linked to recalls, field-alert reports, CAPA, regulator notifications and pharmacovigilance records	Sensitivity, specificity, prevalence-stratified positive and negative predictive value, calibration, net benefit (decision-curve analysis) under a declared loss function, false-escalation rate; reference outcome adjudicated blind to the assigned grade
Lot/batch traceability	Does the framework improve traceability needed for exposure reconstruction?	Before–after implementation study	Complaint intake forms; batch-distribution records; EHR/dispensing data	Lot-capture rate; proportion with analysable exposure window; completeness of product identifiers
Clinical usefulness	Does the framework improve follow-up of	Controlled implementation or interrupted	Hospital systems; pharmacy systems;	Time to follow-up; time to escalation; missed-signal rate; clinician action rate

Validation domain	Core question	Suggested design	Candidate data sources	Primary metrics
	potentially exposed patients?	time-series design	internal safety workflows	
Usability and burden	Can intended users apply the framework consistently and efficiently?	Human-factors evaluation with structured scenarios and live pilot phase	Pharmacists, quality specialists, PV staff, clinicians, patients and carers	Time per case; completion rate; user-rated clarity; reasons for disagreement; patient-comprehensibility and face-validity assessment of the intake fields
Public-health responsiveness	Does the framework help trigger proportionate mitigation without unnecessary disruption?	Mixed-methods implementation evaluation	Recall files; shortage records; communication logs	Time to risk-reducing action; recall/communication timeliness; shortage-related unintended consequences

Table S6. Candidate escalation-threshold variables for PRCS triage

Variable	Suggested role in thresholding
1. Defect evidence — confirmed or highly plausible distributed-batch problem	Automatic escalation trigger
1. Defect evidence — sterility, contamination, wrong product, wrong strength or device non-delivery concern	High-priority trigger
4. Population vulnerability — exposure of a vulnerable population	Priority amplifier
1. Defect evidence — recurrent same-lot or same-defect complaints	Priority amplifier
3. Observed clinical consequence — complaint linked to an adverse event or treatment interruption	Priority amplifier
6. Supply criticality — essential medicine with shortage implications	Requires net-harm assessment before response
2. Exposure and scope — missing lot number with a strong temporal or setting cluster	Escalate despite incomplete traceability
5. Therapeutic margin — narrow therapeutic index, critical-dose product or absence of a monitorable pharmacodynamic marker	Priority amplifier
2. Exposure and scope — quality-system finding indicating probable distributed exposure	High-priority trigger
7. Mechanistic plausibility — plausible defect-to-harm mechanism for the product type (narrow margin, critical care, vulnerable population)	Priority amplifier
3. Observed clinical consequence — serious clinical event compatible with the alleged defect	High-priority trigger
7. Mechanistic plausibility — device, labelling or packaging failure creating an error-forcing condition	High-priority trigger

Supplementary S6. Framework development, decision rules and illustrative application

Part A. Framework development audit trail

The framework was developed by the authors as a process separate from the evidence synthesis described in Supplementary S1. This part records how the categories were generated, so that a reader can distinguish choices forced by the source material from choices that were not. An unvalidated framework still requires a reproducible development process.

Two derivations can be stated on internal grounds. The six clinical-consequence levels were originally a concatenation of two exposure states (A and B) with four severity states (C to F); in the revised framework the exposure content is moved to the exposure gate, so that A–F is a harm scale with an explicit "no harm identified" floor rather than a hybrid of exposure and severity. The five certainty levels of the submitted version were adopted, without acknowledgement, from the WHO–UMC causality categories; the revised framework therefore adopts those categories explicitly and confines the authors' contribution to the two upstream gates that WHO–UMC does not address. Four levels were used for each of those gates, marking not investigated, investigated and negative, partial evidence, and confirmed.

The categories were developed and refined iteratively by the authors through rounds of review. Differences in classification or interpretation were resolved through discussion with reference to the underlying evidence and the intended scope of the framework. The categories were not formally tested against selected historical or hypothetical cases before submission. The framework was not subjected to a formal consensus process or independent external review.

Table S7. Framework-development audit trail

Element	Source material	Design constraint	Alternatives considered and rejected	Rationale for the boundary
PRCS status	Complaint-file and quality-defect obligations (21 CFR 211.198; EU GMP Chapter 8); GVP Module IX.	Must be assignable before defect confirmation and before any adverse-event report exists.	"Candidate signal" (collides with GVP Module IX); "suspected quality defect" (already defined and product-facing).	Boundary set at the plausibility of the alleged defect affecting exposure, sterility, potency, identity, delivery, medication use or treatment continuity.
TRACE — Triage and intake	Complaint-record requirements.	Must capture identifiers before they are lost.	Deferring intake until after investigation.	Evidence perishes; intake must precede confirmation.
TRACE — Reconstruct exposure	Distribution, dispensing and batch-traceability evidence.	Exposure must be graded separately from defect and from causality.	Treating exposure as a component of the causality judgement.	Exposure is a factual state, not a causal judgement.

TRACE — Attribute cautiously	WHO–UMC signal management; established ADR causality practice.	Must not import rechallenge logic.	Naranjo-style additive scoring.	Rechallenge is unavailable for a batch-level defect and the same product from an unaffected lot does not constitute one.
TRACE — Calibrate response	ICH Q9(R1); recall health-hazard evaluation (21 CFR Part 7 Subpart C).	Action must be proportionate to consequence-if-true.	Fixed action thresholds by defect type.	Supply context, clinical margin and availability of substitutes vary.
TRACE — Evaluate and feed back	ICH Q10 CAPA and continual improvement.	Grades must be revisable.	One-time classification at intake.	Re-grading after investigation is expected and is not a failure of the classification.
Clinical-consequence axis A–F	Seriousness criteria; medication-error severity indices.	One ordered dimension only.	Retaining exposure status, process burden, patient experience and event scope on the same axis (as submitted).	States that are not comparable cannot be ordered against one another.
Defect gate d0–d3	Complaint investigation and analytical confirmation practice.	Must separate "not investigated" from "investigated and negative".	A binary confirmed/unconfirmed field.	The two negative states imply different actions: reconstruction versus closure.
Exposure gate e0–e3	Batch-traceability evidence in spontaneous-reporting systems.	Absence of evidence must not be coded as evidence of absence.	A binary exposed/not-exposed field.	e0 (unknown) and e1 (excluded) require different responses.
Attribution gate aNA, a0C, a0U, a1–a4	WHO–UMC causality categories.	No new causality scale.	The bespoke five-level C0–C4 scale used in the submitted version.	Parallel causality terminology has no incremental value in a field already affected by causality-assessment inconsistency.
Qualifier flags S, P, X	Recall scope; shortage, workflow and patient-burden literature.	Scope and burden must not be ordered against clinical harm.	Additional severity levels, or subdivision of Levels C and F.	A cluster is a property of the event set, not a severity of any member of it.
Evidence-eligibility rules	Internal-consistency requirement identified during revision.	Attribution is limited by the upstream evidence, expressed as eligibility criteria rather	Free assignment of all three gates.	Prevents notations such as A/C4, in which the causal axis was used to express certainty about exposure.

		than as arithmetic on the codes.		
Escalation thresholds (Box 2; Table S6)	Risk factors identified across the reviewed sources.	Must be explicit and testable.	Data-derived or calibrated thresholds.	No dataset was available; the thresholds are author-generated, uncalibrated and have no established performance.
Minimum data model (Box 1)	21 CFR 211.198; field-alert triggers; recall hazard elements.	Every field must be traceable to a source or marked as a proposal.	An undifferentiated field list.	Provenance is now shown field by field in Box 1.

Part B. Decision rules for the certainty gates

Defect certainty (d0–d3). d3 requires objective confirmation: analytical testing of the complaint sample or of a retained sample, a documented quality-system failure affecting the batch, or regulatory or manufacturer confirmation of the defect. Visual observation alone, however credible, is d2. A completed investigation that did not confirm the defect is d1, which is not the same as d0, an investigation not performed or not possible.

Exposure certainty (e0–e3). e3 requires a dispensing or administration record concordant with the affected lot or, where lot-level concordance is unavailable, a distribution and use record that excludes unaffected lots. Presence of the affected product in the care setting during the exposure window is e2. e1 requires positive evidence that exposure did not occur, such as quarantine before dispensing; the absence of evidence of exposure is e0, not e1.

Outcome attribution (aNA, a0C, a0U and a1–a4). Graded using an author-adapted operational mapping of the six WHO–UMC causality category labels, as set out in Table S4a. The labels and their ordering are unchanged, but the mapping attaches defect-specific evidence requirements to each grade and links the labels to separate defect and exposure gates that the WHO–UMC system does not contain; it should not be read as the WHO–UMC system applied without modification and has not been validated for defect-to-outcome attribution, with aNA reserved for cases in which no clinical outcome requires attribution or exposure has been excluded, with alternative explanations assessed explicitly and recorded. Dechallenge may contribute where the product is withdrawn and the outcome resolves; rechallenge does not apply, because re-administration of unaffected product does not test the defect.

Evidence-eligibility rules. The three gates are related by evidence eligibility, not by arithmetic: the codes are ordinal labels and are never compared numerically. An attribution of certain (a4) requires a confirmed defect (d3) and documented exposure (e3). An attribution of probable (a3) requires documented exposure (e3) and at least a strongly suspected defect (d2). Where exposure status is unknown (e0), attribution may rise no higher than possible (a2). Where exposure has been excluded (e1), or where no clinical outcome requires attribution, the case is recorded as aNA rather than as a low causality grade. Where harm arises through the indirect supply pathway, so that no patient is exposed to a defective unit and there is no defect object to grade, the defect and exposure gates take the states dNA and eNA; these denote the absence of an object to assess and are distinct from d0 and e0, which denote an object that exists but has not been investigated or established. All four fields are graded per patient; where a source describes a case series,

a representative individual case is graded and the series scope is carried on the S flag, since a single grade cannot simultaneously be an individual grade and an aggregate range. These rules replace the numeric "lower-of" formulation used in the submitted version, which was internally inconsistent: read arithmetically, e0 would have forced attribution to a0 rather than to the a2 ceiling the same text specified. Certainty that a defect exists, certainty that the patient was exposed and certainty that the defect contributed to the outcome remain distinct judgements and are recorded separately.

Part C. Illustrative retrospective application

These are illustrative applications to published cases, not a validation series. Cases were selected because they are already cited in this review and are publicly documented; they were not sampled from a complaint population, no reference standard was applied, no inter-rater exercise was conducted and no performance metric is derived. Their purpose is to demonstrate how the grades resolve and to expose ambiguity in the classification rules.

Table S8. Illustrative retrospective application to published cases

Case (main-text reference)	Illustrative grade	Reasoning	Classification rule that proved ambiguous
Fungal infections associated with contaminated compounded methylprednisolone [38]	F : d3 : e3 : a4 +S(cluster)	Defect confirmed by product testing; exposure documented through lot tracing; fatal outcomes with compatible phenotype and chronology.	Whether the grade describes the individual case or the outbreak. Severity is graded per patient and scope is carried on the S flag.
Oversulfated chondroitin sulfate contamination of heparin [39, 40]	F : d3 : e3 : a3 +S(cluster)	Defect analytically confirmed; exposure documented; mechanism established for the contaminant.	Attribution is graded per patient while the cluster is a property of the set; a single grade cannot represent both.
Diethylene glycol and ethylene glycol contamination of paediatric medicines [36, 37]	F : d3 : e3 : a3 +S(cross-border)	Defect confirmed analytically; exposure documented in some settings and inferred in others; compatible toxicological phenotype.	Exposure grading where dispensing records do not exist: e2 and e3 are separated by a record type that some settings cannot produce.
Nitrosamine impurity exposure in valsartan products [34]	U : d3 : e3 : aNA	Defect confirmed and exposure is reconstructible from dispensing data. Because the incomplete outcome window cannot distinguish no effect from inadequate ascertainment, the case is graded U (not B9: outcome not adequately assessed within follow-up, with no basis for attribution (aNA). A complete outcome window would require reassessment at both gates.	Long-latency outcomes: the consequence axis records what has been observed, so an incomplete outcome window is not distinguishable from an absence of effect. Because no mechanism-matched outcome had been observed within the available follow-up, there was no object for attribution and the case takes aNA rather than a low causality grade; a later observed outcome would require regrading.

Norepinephrine supply disruption [35]	F : dNA : eNA : aNA +S(multi-site) +P	The framework presumes a defective unit. In recall- or shortage-mediated harm, no patient is exposed to one, so the defect and exposure gates are not applicable (dNA, eNA), unlike d0 and e0, which indicate an assessable object that was not investigated. Attribution is therefore aNA and the case is classified as an indirect pathway.	Whether indirect supply-mediated harm belongs in a notation built around a defective unit. dNA and eNA mark the absence of an assessable object and are distinct from d0 and e0.

Sources

Sources are numbered S1 to S25 so that they can be cited from the supplementary text. Where a source also appears in the numbered reference list of the main manuscript, the main-text reference number is given in brackets. Sources S18 to S21 support statements made in Supplementary S5 and had no numbered citation in the submitted version. Sources S22 to S25 were added at revision and are also cited in the main reference list.

- *S1. US eCFR, 21 CFR 211.198, Complaint files. Complaint-record content and investigation documentation.*
- *S2. US eCFR, 21 CFR 314.81, Other postmarketing reports. NDA field-alert reporting requirements.*
- *S3. FDA, Field Alert Reports page. Current operational summary of field-alert purpose and triggers.*
- *S4. FDA, Field Alert Report Submission: Questions and Answers Guidance for Industry (2021).*
- *S5. US eCFR, 21 CFR Part 7 Subpart C, Recalls. Recall policy, health-hazard evaluation, classification and strategy.*
- *S6. FDA, FDA Adverse Event Monitoring System (AEMS) page. Consolidated post-marketing safety platform and relationship to legacy systems (from 2026).*
- *S7. FDA, FDA Adverse Event Reporting System (FAERS) database page. Scope and coverage of the legacy FAERS database.*
- *S8. ICH E2E, Pharmacovigilance Planning (Step 4, 2004). Safety specification; identified/potential risks; missing information; routine/additional pharmacovigilance.*
- *S9. ICH Q10, Pharmaceutical Quality System (Step 4, 2008). Complaint/CAPA/lifecycle integration.*
- *S10. EMA page for ICH Q9(R1), Quality risk management. Current EU scientific-guideline page confirming lifecycle application of quality risk management.*
- *S11. EU GMP Chapter 8, Complaints, Quality Defects and Product Recalls. Chapter 8 principles, investigation, authority notification, CAPA and recall expectations.*
- *S12. WHO fact sheet, Substandard and falsified medical products (2024). Burden, harms, GSMS description and proportionate-response principles.*
- *S13. WHO, Global Surveillance and Monitoring System for Substandard and Falsified Medical Products (2017). Foundational GSMS report.*
- *S14. WHO, GSMS activity report, August 2017–December 2021 (2024 publication). Recent cross-border incident activity report.*
- *S15. UMC, What is a signal? Signal definition and non-equivalence with causality.*

- *S16. UMC, Signal management at UMC. Five-phase signal-management process and communication pathway.*
- *S17. UMC, About VigiBase. Global database scale and current description.*
- *S18. CIOMS Working Group VIII, Practical Aspects of Signal Detection in Pharmacovigilance. Operational metrics, quality assurance and linkage to broader pharmacovigilance programmes.*
- *S19. EQUATOR/STROBE statement page. Reporting standard for observational studies.*
- *S20. EQUATOR/RECORD statement page. Reporting standard for studies using routinely collected health data.*
- *S21. FDA, Framework for FDA's Real-World Evidence Program (2018). RWD/RWE definitions and relevance of observational studies and routinely collected data.*
- *S22. UMC, The Use of the WHO–UMC System for Standardised Case Causality Assessment. Definitions of the six causality categories used at the outcome-attribution gate. [Main-text reference 72.]*
- *S23. ICH E2A, Clinical Safety Data Management: Definitions and Standards for Expedited Reporting (Step 4, 1994). Standard seriousness criteria. [Main-text reference 69.]*
- *S24. ICH E2D(R1), Post-Approval Safety Data (Step 4, 15 September 2025). Current post-approval definitions and case-management standards. [Main-text reference 70.]*
- *S25. EMA GVP Annex I — Definitions (Rev 5, 2024). Current EU definitions, including seriousness. [Main-text reference 71.]*
